# Difference of Admission Neutrophil Gelatinase-Associated Lipocalin Concentration Between Patients Developing and Not Developing Acute Kidney Injury or Need for Acute Dialysis: An Ancillary Individual-Study Data Meta-Analysis (INDICATE–AKI)

**DOI:** 10.1016/j.xkme.2026.101280

**Published:** 2026-02-02

**Authors:** Annemarie Albert, Louisa Blume, Salvatore Di Somma, Mina Hur, Rinaldo Bellomo, Prasad Devarajan, Tobias Breidthardt, Fabrice Camou, Sidney Chocron, Dinna Cruz, Hilde RH. de Geus, Kent Doi, Zoltan H. Endre, Mercedes Garcia-Alvarez, Michael Haase, Anja Haase-Fielitz, Peter Buhl Hjortrup, Georgios Karaolanis, Cemil Kavalci, Hanah Kim, Sebastian Lange, Philipp Lauten, Paolo Lentini, Christoph Liebetrau, Miklós Lipcsey, Johan Mårtensson, Christian Müller, Serafim Nanas, Thomas L. Nickolas, John W. Pickering, Chrysoula Pipili, Claudio Ronco, Guillermo Rosa-Diez, Azrina Md Ralib, Karina Soto, Philipp Stieger, Antonia Zapf, Rüdiger C. Braun-Dullaeus, Christian Albert

**Affiliations:** 1 Department of Nephrology, Central Clinic Bad Berka, Bad Berka, Germany; 2 University Clinic for Cardiology and Angiology, Otto-von-Guericke University Magdeburg, Magdeburg, Germany; 3 Department of Neonatology, Charité University Medicine Berlin, Berlin, Germany; 4 Postgraduate School of Emergency Medicine, Faculty of Medicine and Psychology, University La Sapienza of Rome, Rome, Italy; 5 Department of Laboratory Medicine, Konkuk University School of Medicine, Seoul, Republic of Korea; 6 Department of Intensive Care, The Austin Hospital, Melbourne, Australia; 7 Centre for Integrated Critical Care, The University of Melbourne, Melbourne, Australia; 8 Division of Nephrology and Hypertension, Cincinnati Children's Hospital, University of Cincinnati, Ohio, USA; 9 Departments of Internal Medicine, Nephrology and Cardiology, University Hospital Basel, Basel, Switzerland; 10 Service de réanimation médicale, Hôpital Saint-André, Centre Hospitalier Universitaire de Bordeaux, Bordeaux, France; 11 Department of Thoracic and Cardio-Vascular Surgery, University Hospital Jean Minjoz, Besançon, France; 12 Division of Nephrology and Hypertension, University of California, San Diego, California, USA; 13 Department of Intensive Care, Erasmus University Medical Center, Rotterdam, The Netherlands; 14 Department of Emergency and Critical Care Medicine, The University of Tokyo, Hongo, Bunkyo, Tokyo, Japan; 15 Department of Nephrology, Prince of Wales Hospital and Clinical School, University of New South Wales, Randwick, Sydney, Australia; 16 Department of Anesthesiology, Hospital de la Santa Creu i Sant Pau, Barcelona, Spain; 17 Department of Nephrology and Hypertension, Hannover Medical School, Hannover, Germany; 18 Medical Faculty, Otto-von-Guericke University Magdeburg, Magdeburg, Germany; 19 Department of Cardiology, Immanuel Diakonie Bernau, Heart Center Brandenburg, Brandenburg Medical School Theodor Fontane (MHB), Bernau, Germany; 20 Institute of Social Medicine and Health Systems Research, Otto-von-Guericke University Magdeburg, Magdeburg, Germany; 21 Department of Intensive Care, Copenhagen University Hospital—Rigshospitalet, Copenhagen, Denmark; 22 Department of Cardiothoracic Anaesthesia and Intensive Care, The Heart Center, Copenhagen University Hospital—Rigshospitalet, Copenhagen, Denmark; 23 Vascular Unit, First Department of Surgery, “Laiko” General Hospital, Medical School, National and Kapodistrian University of Athens, Athens, Greece; 24 Emergency Department, Faculty of Medicine, Baskent University, Ankara, Turkey; 25 Department of Cardiology and Intensive Care Medicine, Central Clinic Bad Berka, Bad Berka, Germany; 26 Medical Faculty, Philipps University of Marburg, Marburg, Germany; 27 Department of Nephrology and Dialysis, San Bassiano Hospital, Bassano del Grappa, Italy; 28 Center for Cardiology and Angiology, Agaplesion Bethanien Krankenhaus, Frankfurt, Germany; 29 Department of Cardiology, Kerckhoff Clinic, Bad Nauheim, Germany; 30 CIRRUS, Hedenstierna laboratory, Anaesthesiology and Intensive care, Department of Surgical Sciences, Uppsala University, Uppsala, Sweden; 31 Section of Anaesthesia and Intensive Care Medicine, Department of Physiology and Pharmacology, Karolinska Institutet, Stockholm, Sweden; 32 First Critical Care Department, 'Evangelismos' General Hospital, National and Kapodistrian University of Athens, Athens, Greece; 33 Department of Medicine, Division of Nephrology, Columbia University, New York, New York, USA; 34 Department of Emergency Medicine, Christchurch Hospital, Christchurch, New Zealand; 35 Department of Medicine, University of Otago Christchurch, Christchurch, New Zealand; 36 Medical Faculty, University of Padova and Department of Nephrology, Dialysis & Transplantation, International Renal Research Institute Vicenza (IRRIV), San Bortolo Hospital, Vicenza, Italy; 37 Department of Nephrology, Dialysis and Transplantation, Hospital Italiano de Buenos Aires, Buenos Aires, Argentina; 38 Department of Anaesthesiology and Intensive Care, International Islamic University Malaysia, Kuantan, Pahang, Malaysia; 39 Department of Nephrology, Hospital Fernando Fonseca, Lisbon, Portugal; 40 Department of Medical Biometry and Epidemiology, University Medical Center Hamburg-Eppendorf, Hamburg, Germany

**Keywords:** Acute kidney injury, neutrophil gelatinase-associated lipocalin, NGAL, subclinical AKI, meta-analysis, renal replacement therapy, renal risk assessment, clinical decision making

## Abstract

**Rationale & Objective:**

Patients admitted to the emergency department, the intensive care unit (ICU), and after cardiac surgery are at increased risk of developing adverse kidney events. Assessment of neutrophil gelatinase-associated lipocalin (NGAL) may facilitate renal risk prediction. However, the difference in NGAL-concentrations at admission in patients developing and not developing adverse events is unclear.

**Study Design:**

An ancillary meta-analysis to a previous systematic review and meta-analysis using reanalyzed individual study-data from prospective clinical studies to compare NGAL concentrations measured using clinical laboratory platforms at patient admission. The study followed the Preferred Reporting Items for a Systematic Review and Meta-analysis of Individual Participant Data guideline.

**Setting & Study Populations:**

Studies of adults investigating acute kidney injury (AKI) of all stages, severe AKI (stage injury or failure), and acute initiation of renal replacement therapy (RRT) in the setting of cardiac surgery, emergency department, or intensive care unit using either urinary or plasma NGAL concentrations measured on clinical laboratory platforms.

**Selection Criteria for Studies:**

Data inclusion was limited to the individual study-level data from the predecessor study.

**Data Extraction:**

This study used individual study-level data acquired using the protocol of a previous study, which was accomplished by individual authors’ reassessment of their study data.

**Analytical Approach:**

Classification of AKI was harmonized among studies. Prespecified data comparison was performed for urine and plasma specimens for the outcome measures AKI, severe AKI, and acute RRT-initiation. Random effects meta-analyses were performed using the inverse variance method and the DerSimonian and Laird heterogeneity estimator.

**Results:**

In total, 30 data sets from 26 studies were included. The estimated mean difference of urine NGAL concentrations was 125 (95% CI, 57.33-193.54) ng/mL for AKI, 317 (95% CI, 134.95-499.82) ng/mL for severe AKI, and 331 (95% CI, 71.36-592.06) ng/mL for RRT. For plasma NGAL concentrations, the estimated mean differences were 86.04 (95% CI, 51.74-120.34) ng/mL for AKI, 150.52 (95% CI, 80.27-220.76) ng/mL for severe AKI, and 129.83 (95% CI, 79.03-180.63) ng/mL for RRT. There were subgroup differences for the clinical setting, but not for the use of the urine output criterion. Multiple studies showed elevated NGAL concentrations in patients without serum creatinine concentration-based AKI, likely identifying patients with suspected AKI stage 1S (subclinical AKI).

**Limitations:**

Imperfect harmonization of data across studies because of their original protocols.

**Conclusions:**

NGAL concentration differences may facilitate identification of patients at risk of AKI or with suspected AKI stage 1S at admission. Heterogeneity and variability across studies, specimen types, and settings emphasize the importance of interpreting NGAL values within the specific clinical context and patient population.

**Study Registration:**

The International Database of Prospectively Registered Systematic Reviews reg. no.: CRD42016042735. Version of Record 1.2.

## Plain-language Summary

Patients admitted to the intensive care unit, the emergency department, or following cardiac surgery are at increased risk of acute kidney injury (AKI). Neutrophil gelatinase-associated lipocalin (NGAL) is a biomarker that may help stratify AKI risk. This meta-analysis pooled and reanalyzed data from prospective studies measuring NGAL levels at patient admission and systematically compared them in those patients who developed AKI or required renal replacement therapy with those who did not. Higher NGAL levels were found to be associated with unfavorable outcomes. However, variability across studies and settings was observed. Interestingly, some patients showed elevated NGAL levels despite not being affected by serum creatinine-based AKI, suggesting NGAL levels may reflect subclinical AKI (stage 1S). These findings highlighted the need to interpret NGAL concentrations contextually within clinical settings.

## Introduction

Acute kidney injury (AKI) is a common complication among patients admitted to the emergency department (ED), those in the intensive care unit (ICU), or those undergoing cardiac surgery (CS) procedures, and it is associated with significantly higher rates of morbidity and mortality.^1^

Markers of kidney tubular damage may enable refined AKI risk assessment when measured at the time of patient admission.^2^ Level of neutrophil gelatinase-associated lipocalin (NGAL) is a kidney biomarker studied in depth for its ability to indicate early structural damage and provide insight into patient kidney disease prognosis in acute settings.^3^, ^4^, ^5^

In the primary assessment of the present data set, we recently derived cutoff concentrations to rule out and rule in patients at increased risk of developing AKI, severe AKI, or the need for acute renal replacement therapy (RRT) initiation by reassessing 30 data sets from 26 prospective studies on NGAL’s predictive capabilities.^6^

Additional data acquired with the previous data set enable the derivation of summary distributions of NGAL in patients at the time of ED or ICU admission, which no previous study has reported on. As an adjunct to clinical decision-making, however, observed trends might reveal specific patterns and support clinicians in assessing the degree of elevation of NGAL concentrations as an early indicator of kidney risk.

In the present study, we therefore used the data set acquired for the previous investigation,^6^ to subsequently examine whether NGAL concentrations sampled at admission to the ED or ICU would differ between patients who would go on to develop serum creatinine (SCr)-based AKI, severe AKI, or require acute RRT initiation versus those who would not develop such adverse outcomes in the clinical course. To perform this ancillary analysis of a prior meta-analysis, we used individual study data previously obtained from prospective clinical studies that measured the concentration of NGAL in urine or plasma using clinical laboratory platforms to predict AKI, severe AKI, or the need for RRT.^6^

We hypothesized that differences in NGAL concentrations exist for those with and without the respective outcome measure of interest.

## Methods

### Overview and Relation With a Previous Meta-analysis

This is an ancillary analysis of a previous meta-analysis that focused on the derivation of NGAL cutoff concentrations for the prediction of AKI, severe AKI, and the necessity of acute RRT initiation.^6^ The analysis was restricted to diagnostic test studies of adult humans investigating the prediction of AKI or RRT necessity in the setting of critical illness related to CS or admission to ED or ICU using either urine or plasma NGAL concentration measured exclusively on clinical laboratory platforms (Supplementary Item S1, Table S1). Studies using laboratory research methods, such as enzyme-linked immunosorbent assays for the detection of NGAL, were not considered. The derivation of prespecified primary NGAL indices included, among others,^6^ the cutoff concentration at the maximum Youden index and the summary receiver operator characteristic (ROC) curve values. The primary study results,^6^ and further methodological assessments were previously reported in detail.^7^

The study aims, search strategy, data extraction, and data synthesis were registered with the International Database of Prospectively Registered Systematic Reviews (http://www.crd.york.ac.uk/prospero, reg.-no.: CRD42016042735). Version of Record 1.2, including the aims of the present analysis, was filed on January 6, 2025. The Preferred Reporting Items for a Systematic Review and Meta-Analysis of Individual Participant Data were adhered to.^8^

The extensive methodology of data sourcing, search strategy, and the process of study selection, data extraction, and quality assessment are available elsewhere.^6^

In brief, this individual-study-data meta-analysis used standardized custom-made data sheets requesting data reanalysis on the patient level by the authors of the original diagnostic test studies. Authors of relevant studies were requested to exclude patients with known AKI or RRT at admission, or with NGAL measurement within 24 hours before diagnosis of AKI or RRT initiation, from their summary, with the intention to provide a predictive rather than affirmative overview of NGAL’s performance in distinguishing patients who would go on to develop AKI or need RRT initiation from those who did not.^6^ The study flowchart is provided in Fig S1.

### Harmonized AKI Classification Criteria

Ensuring consistency among studies, this individual-study data-based meta-analysis used reanalyzed uniform classification for AKI according to a standardized consensus definition classified by severity according to the Risk, Injury, Failure, Loss of kidney function, and End-stage kidney disease (RIFLE) criteria based on increases in SCr concentrations from their respective baseline within 7 days, as well as urine output criteria (UOC) where available.^9^

### Outcome Measures

Corresponding to the predefined outcome measures in the primary investigation,^6^ the present meta-analysis used 3 outcome measures, with calculations performed separately for urine and plasma specimens: AKI (of all stages), severe AKI defined as RIFLE stages I (injury) or F (failure), and acute initiation of RRT.

### Aim of the Present Individual-study-data Meta-analysis

For this complementary ancillary analysis, the primary aim was the assessment of the mean difference (MD) of admission NGAL concentrations in patients developing and those not developing an adverse outcome measure, predefined in the previous paragraph. In detail, this meta-analysis assessed the MD of (1) patients developing AKI versus those without AKI; (2) patients developing severe AKI versus those without AKI; and (3) patients needing acute RRT initiation versus those without RRT initiation. To enable this, the original protocol previously requested the mean NGAL concentrations and corresponding standard deviations (SDs) of these specific patient groups sampled at the time of admission to 3 settings, namely ED, ICU, or after CS from all participating studies.^6^ An excerpt of the original data request sheet is available in Table S2.

Complementary to the previous study, subgroup analyses were performed to assess whether differences in the mean NGAL concentrations exist for studies considering the UOC for AKI classification and those not considering the UOC (Item S2.4). In the main study, results of subgroup analysis for the clinical setting are provided.

### Definition of Subclinical AKI in the Post Hoc Analysis

Following propositions by Ostermann et al,^2^ we defined subclinical AKI (AKI stage 1S) as elevated NGAL concentrations above the NGAL cutoff concentration to predict AKI defined by each study’s calculated maximum Youden index in the absence of an AKI-defining SCr concentration increase (RIFLE−status).^2^ Alternatively, the analysis was performed using the meta-analyzed cutoff concentration,^6^ derived from the summary ROC curve value to predict AKI. Elevated NGAL concentrations above the threshold were attributed to defining NGAL-positivity and thus AKI stage 1S.^2^

### Statistical Analyses

Meta-analyses were conducted as prespecified, separately for urine and plasma specimens for the baseline NGAL concentration at patients’ admission for each outcome measure.

The number of patients in each setting, the arithmetic mean (mean) value, and the SD of the mean were used to perform the meta-analysis. Studies had to be excluded from individual analyses if respective data on mean value or SD were missing or in the case of individual event rates of N ≤ 1, as then no SD is calculable.

To compare continuous variables, MDs and their corresponding SDs were used, applying the inverse variance method with a random-effects model to account for potential heterogeneity across studies. A positive result implies that the group with the adverse event is associated with a higher NGAL concentration at admission. Within-subgroup differences were assessed using the χ^2^ test. Because of the suspected clinical and methodological heterogeneity, the between-study variance (τ^2^) was estimated using the DerSimonian and Laird estimators.^10^ The 95% confidence intervals (CIs) for τ^2^ were calculated using Jackson’s method, providing reliable interval estimates in the presence of heterogeneity. The I^2^ statistic was calculated to quantify the percentage of total variability in the effect estimates attributable to heterogeneity rather than sampling error. Values of I^2^ were interpreted as suggested by Higgins et al.^11^

### Software

Statistical analyses were conducted using the R environment for statistical computing (R Foundation for Statistical Computing) with the meta package version 8.0-1.

## Results

### Inclusion of Studies

In total, 30 data sets from 26 observational studies were included.^12^, ^13^, ^14^, ^15^, ^16^, ^17^, ^18^, ^19^, ^20^, ^21^, ^22^, ^23^, ^24^, ^25^, ^26^, ^27^, ^28^, ^29^, ^30^, ^31^, ^32^, ^33^, ^34^, ^35^, ^36^, ^37^ Twelve studies provided data on urine NGAL concentration,^12^, ^13^, ^14^, ^15^, ^16^, ^17^, ^18^, ^19^, ^20^, ^21^, ^22^, ^23^ and 18 studies on plasma NGAL concentrations,^20^, ^21^, ^22^, ^23^, ^24^, ^25^, ^26^, ^27^, ^28^, ^29^, ^30^, ^31^, ^32^, ^33^, ^34^, ^35^, ^36^, ^37^ whereas 4 studies provided data on NGAL concentrations in both urine and plasma.^20^, ^21^, ^22^, ^23^ Several studies with individual event rates of N ≤ 1 for individual outcome measures were left out of the respective analysis because no SD was estimable,^18^, ^19^, ^20^^,^^24^, ^25^, ^26^, ^27^, ^28^ or because of missing data.^12^^,^^37^

Characteristics of included studies are reported in Table 1^12^, ^13^, ^14^, ^15^, ^16^, ^17^, ^18^, ^19^, ^20^, ^21^, ^22^, ^23^, ^24^, ^25^, ^26^, ^27^, ^28^, ^29^, ^30^, ^31^, ^32^, ^33^, ^34^, ^35^, ^36^, ^37^, Table S3 and Item S2.1. Additional results are available in Supplementary Material S2.

**Table 1:** Baseline Characteristics of Individual Studies Separated for Urine and Plasma Data

Reference	Setting	UOC	Laboratory Platform	Mean NGAL Concentrations at Admission to ED, ICU, or after CS, ng/mL (SD)					Timing of NGAL Measurements
				No-AKI	AKI	RIFLE Injury or Failure	No-RRT	RRT	
**Urine NGAL concentration, ng/mL**									
Mårtensson et al,^20^ 2015	ICU	YES	ARCHITECT	750.00 (1,520.00)	182.70 (300.30)	—	671.30 (1,418.00)	22.60 (NA)	< 24 h of AKI diagnosis
Pipili et al,^15^ 2014	ICU	YES	ARCHITECT	300.65 (257.83)	576.97 (478.64)	934.70 (98.50)	164.39 (540.66)	780.84 (186.56)	ICU admission
Hjortrup et al,^22^ 2015	ICU	NO	BIOPORTO	883.10 (2,208.40)	2,812.00 (5,129.00)	3,940.50 (6,197.60)	1,353.90 (3,316.80)	1,793.20 (3462.50)	4 h after ICU admission
de Geus et al,^21^ 2011	ICU	YES	TRIAGE	271.00 (625.00)	1,080.00 (1,477.00)	1,643.00 (1,770.00)	323.00 (720.00)	1,659.00 (1928.00)	ICU admission
Dai et al,^18^ 2015	ICU	YES	TRIAGE	147.03 (10.12)	243.27 (19.03)	287.22 (144.75)	191.00 (119.00)	—	ICU admission, daily
Ralib et al,^23^ 2014	ICU	NO	TRIAGE	333.14 (526.49)	144.15 (182.22)	144.15 (182.22)	333.14 (526.49)	144.15 (182.22)	ED, ICU, 2, 4, 8, and 16 h; 2, 4, and 7 d
Nickolas et al,^13^ 2012	ED	NO	ARCHITECT	98.96 (269.06)	399.02 (944.48)	731.70 (1,223.94)	174.04 (552.75)	611.55 (826.75)	within 12 h
Liebetrau et al,^14^ 2013	CS	YES	ARCHITECT	37.73 (120.52)	48.24 (82.74)	80.13 (99.34)	34.96 (91.86)	285.53 (395.39)	4h after ICU admission
Karaolanis et al,^19^ 2015	CS	NO	ARCHITECT	31.30 (92.90)	45.00 (105.90)	—	—	—	3 h after Surgery
Varela et al,^12^ 2015	CS	YES	ARCHITECT	67.20 (156.80)	112.00 (232.00)	83.90 (75.50)	74.80 (174.00)	177.80 (NA)	6 h after ICU admission
Garcia-Alvarez et al^17^, 2015	CS	NO	ARCHITECT	194.40 (490.18)	390.04 (836.80)	500.66 (1,095.18)	258.42 (643.43)	534.69 (910.33)	ICU admission
Haase et al,^16^ 2013	CS	YES	ARCHITECT	65.33 (159.04)	288.07 (427.52)	431.50 (545.72)	67.54 (157.52)	424.36 (549.54)	6 h after the beginning of CPB

Reference	Setting	UOC	Laboratory Platform	Mean NGAL concentrations at admission to ED, ICU, or after CS, ng/mL (SD)					Timing of NGAL measurements
				No-AKI	AKI	RIFLE Injury or Failure	No-RRT	RRT	
***Plasma NGAL***									
Cruz et al,^27^ 2010	ICU	YES	TRIAGE	132.00 (144.00)	170.00 (142.00)	248.00 (255.00)	140.00 (145.00)	118.00 (NA)	< 24 h of AKI diagnosis, daily
Camou et al,^30^ 2013	ICU	YES	TRIAGE	191.40 (185.40)	481.80 (269.80)	483.70 (272.80)	363.60 (263.00)	540.40 (280.50)	2, 24, and 48 h after ICU admission
de Geus et al,^21^ 2011	ICU	YES	TRIAGE	179.00 (143.00)	329.00 (268.00)	419.00 (327.00)	194.00 (168.00)	330.00 (191.00)	ICU admission
Katagiri et al,^24^ 2013	ICU	NO	TRIAGE	122.50 (101.50)	243.30 (184.10)	325.80 (181.70)	—	—	< 24h of AKI diagnosis
Kim et al,^25^ 2013	ICU	NO	TRIAGE	387.10 (356.60)	258.20 (188.60)	284.30 (207.60)	203.00 (233.40)	294.00 (NA)	< 24 h of AKI diagnosis
Lentini et al,^34^ 2012	ICU	NO	TRIAGE	213.00 (226.00)	581.00 (306.00)	764.00 (233.00)	463.00 (295.00)	784.00 (206.00)	4 h after ICU admission
Mårtensson et al,^20^ 2015	ICU	YES	TRIAGE	136.80 (128.50)	149.00 (120.50)	—	141.20 (126.60)	60.00 (NA)	< 24 h of AKI diagnosis
Pickering and Endre,^35^ 2013	ICU	YES	TRIAGE	77.16 (150.14)	144.44 (153.04)	144.44 (153.04)	76.62 (148.65)	194.50 (175.44)	ICU admission, 12 h, 24 h, daily
Ralib et al,^23^ 2014	ICU	NO	TRIAGE	207.97 (225.67)	206.75 (160.77)	206.75 (160.77)	205.39 (219.72)	248.00 (239.00)	ED, ICU, 2, 4, 8, and 16 h; 2, 4, 7d
Hjortrup et al,^22^ 2015	ICU	NO	BIOPORTO	410.50 (356.30)	618.70 (533.30)	708.30 (659.90)	423.70 (348.20)	926.40 (772.60)	4 h after ICU admission
Breidthardt et al,^26^ 2012	ED	NO	TRIAGE	107.90 (71.30)	136.40 (84.20)	132.20 (75.20)	—	—	every 6 h, < 24 h of AKI diagnosis
Di Somma et al,^29^ 2013	ED	YES	TRIAGE	132.80 (133.00)	206.30 (244.60)	287.80 (223.60)	134.60 (137.90)	537.00 (277.10)	0, 6, 12, 24, 48, and 72 h
Kavalci et al,^37^ 2014	ED	NO	TRIAGE	—	600.76 (345.85)	515.37 (368.78)	506.44 (351.59)	724.11 (301.99)	ED admission, 6 h after admission
Soto et al,^33^ 2013	ED	NO	TRIAGE	94.02 (65.12)	205.62 (208.91)	321.90 (279.66)	123.98 (135.21)	210.70 (141.36)	0, 6, 12, 24, and 48 h
Doi et al,^36^ 2013	CS	NO	TRIAGE	104.01 (106.44)	141.52 (87.33)	133.39 (82.81)	107.88 (104.54)	176.50 (65.06)	0, 2, 4, 12, 24, 36, and 60 h after ICU arrival
Perrotti et al,^32^ 2015	CS	NO	TRIAGE	192.97 (170.70)	316.02 (243.06)	426.06 (318.64)	228.00 (205.00)	358.00 (72.00)	6 h after the end of surgery
Lipcsey et al,^31^ 2014	CS	NO	TRIAGE	116.00 (64.00)	129.00 (80.00)	137.00 (103.00)	120.00 (70.00)	129.00 (86.00)	ICU admission
Park et al,^28^ 2015	CS	NO	TRIAGE	91.30 (44.60)	126.10 (90.00)	179.20 (154.40)	113.00 (77.70)	—	ICU admission

All numbers denote urine or plasma NGAL concentrations in ng/mL units with their means and standard deviation (SDs).

Abbreviations: AKI, acute kidney injury; CS, cardiac surgery; ED, emergency department; ICU, intensive care unit; NGAL, neutrophil gelatinase-associated lipocalin; UOC, consideration of urine output criterion for AKI; RIFLE, risk, injury, failure, loss of kidney function, end-stage kidney disease classification; RRT, renal replacement therapy.

### Quality Assessment

The funnel plots for the present data do not exhibit obvious asymmetry and hence provide no evidence for systematic selection bias or indication of small-study effects.^38^ The in-depth interpretation of funnel plots is outlined in Item S2.2. The quality assessments are available in Item S2.3.

### Distribution of Studies’ Mean Urine and Plasma NGAL Concentrations

**Figure 1:**
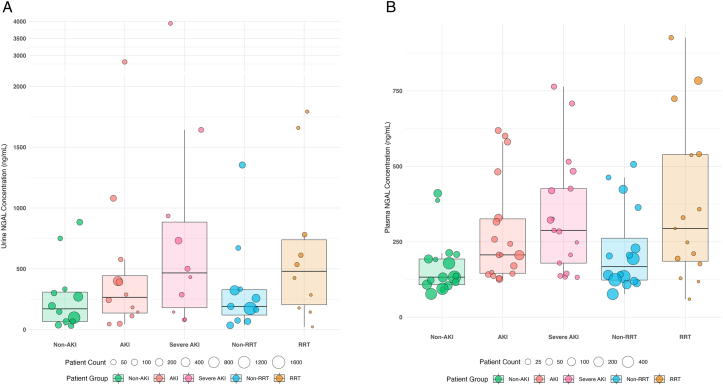
(A and B) Unweighted descriptive distribution of studies’ mean urine (A) and plasma (B) NGAL concentrations (in ng/mL) according to studies’ patients with and without the outcome measures AKI, severe AKI, or RRT. Each bubble is representative of the group sample size of each study. Boxes represent the median (25-75^th^ IQR) of all available studies’ mean urine and plasma NGAL concentrations; whiskers represent ±1.5× multiple of IQR. Abbreviations: AKI, acute kidney injury; IQR, interquartile range; NGAL, neutrophil gelatinase-associated lipocalin; RRT, renal replacement therapy.

To obtain an overview of the collected raw individual-study NGAL concentration data, we derived unweighted descriptive statistics of the mean urine and plasma NGAL concentrations at admission, separately for the outcome measures and specimen, summarized in Fig 1 and Table 1^12^, ^13^, ^14^, ^15^, ^16^, ^17^, ^18^, ^19^, ^20^, ^21^, ^22^, ^23^, ^24^, ^25^, ^26^, ^27^, ^28^, ^29^, ^30^, ^31^, ^32^, ^33^, ^34^, ^35^, ^36^, ^37^ Mean NGAL concentrations increased incrementally with the severity of AKI or RRT versus non-RRT. The number of outliers was low and represented studies with low patient numbers.^22^

### Evidence Synthesis

**Figure 2:**
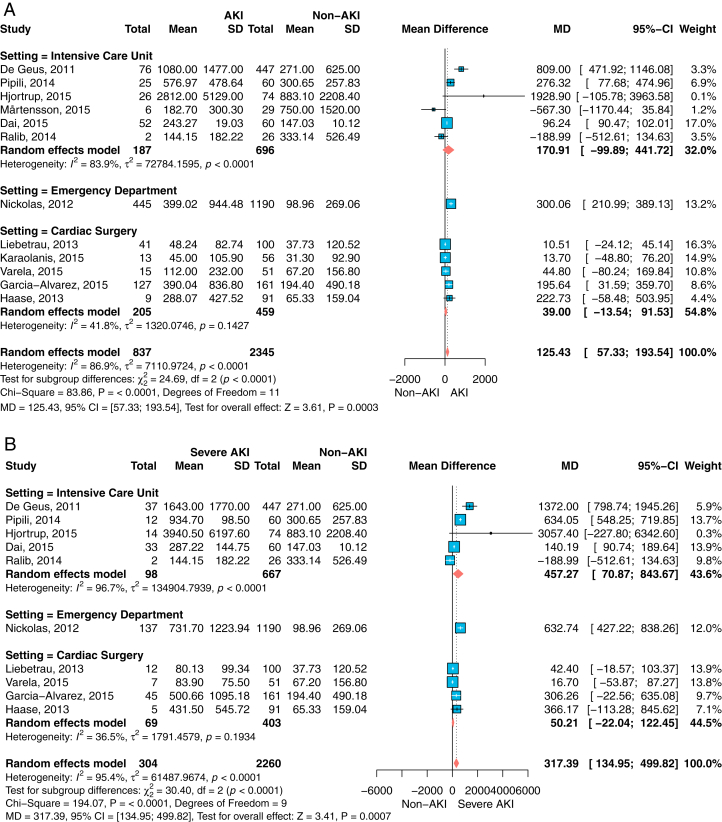

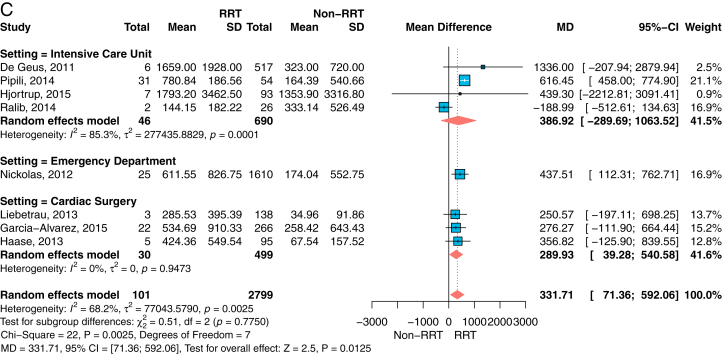
Forest plots for the mean difference of urine NGAL concentrations and standard deviation (SD) for those patients with the outcome measure AKI (A), severe AKI (B, defined as RIFLE stages injury or failure), or (C) RRT versus those without AKI or RRT, respectively, grouped by setting. For each study, the inverse variance weights—in terms of percentage contribution to the overall estimate—are provided. Overall summary and subgroup estimates are presented as the mean difference (MD) with 95% confidence interval (CI). Units for urine NGAL concentrations apply as ng/mL. Abbreviations: AKI, acute kidney injury; NGAL, neutrophil gelatinase-associated lipocalin; RIFLE, risk, injury, failure, loss of kidney function, end-stage kidney disease classification; RRT, renal replacement therapy.

**Figure 3:**
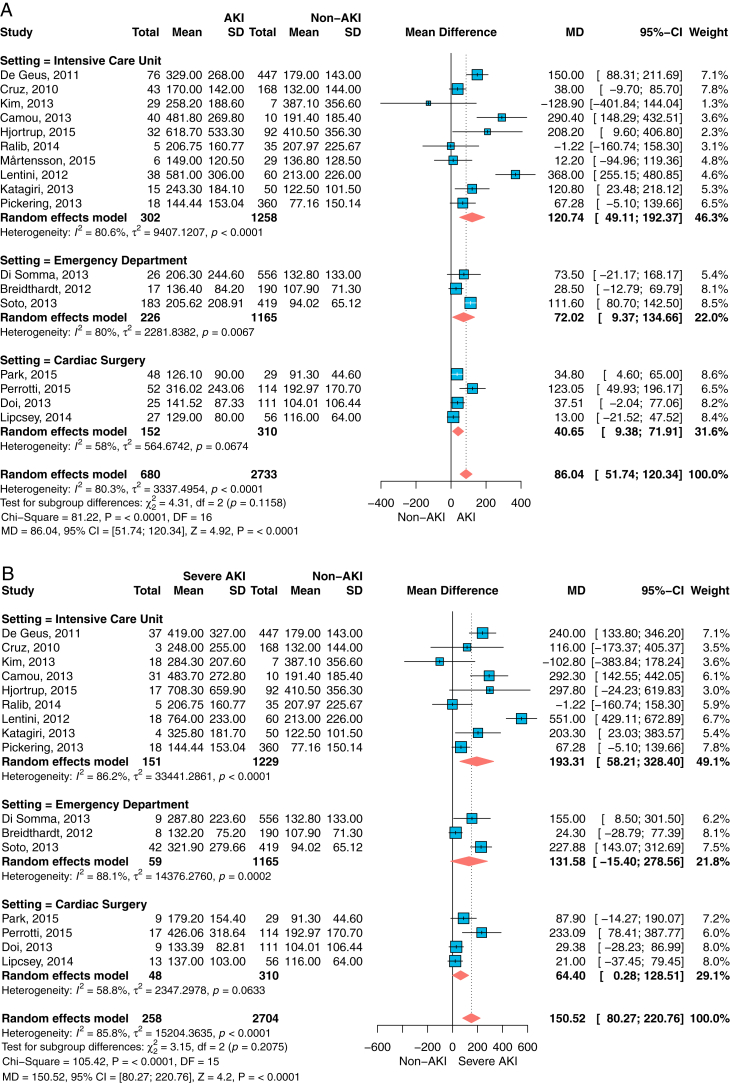

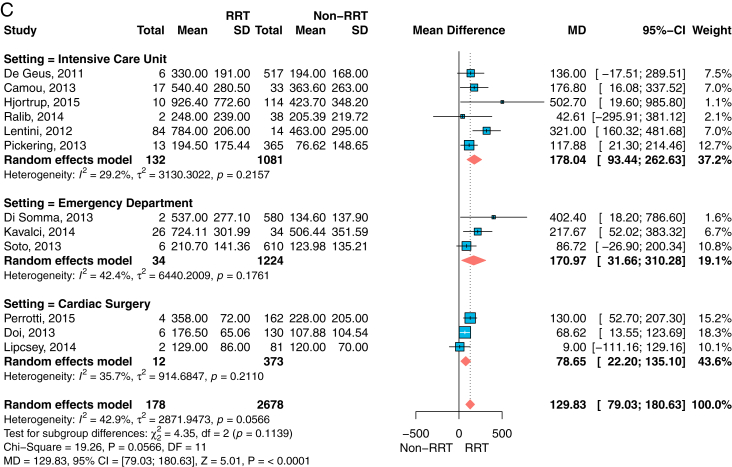
Forest plots for the mean difference of plasma NGAL concentrations and standard deviation (SD) for those patients with the outcome measure AKI (A), severe AKI (B, defined as RIFLE stages injury or failure, or (C) RRT versus those without AKI or RRT, respectively, grouped by setting. For each study, the inverse variance weights—in terms of percentage contribution to the overall estimate—are provided. Overall summary and subgroup estimates are presented as the mean difference (MD) with 95% confidence interval (CI). Units for plasma NGAL concentrations apply as ng/mL. Abbreviations: AKI, acute kidney injury; NGAL, neutrophil gelatinase-associated lipocalin; RIFLE, risk, injury, failure, loss of kidney function, end-stage kidney disease classification; RRT, renal replacement therapy.

The data obtained were sufficient to perform 6 meta-analyses as outlined in the aims and methodology: 3 for urine NGAL concentrations (Fig 2A-C) and 3 for plasma NGAL concentrations (Fig 3A-C). For each specimen type, analyses were conducted for the outcome measures AKI, severe AKI, and RRT. The estimates for the MD of NGAL, as well as patient and event numbers, are shown within their respective figures. Meta-analysis for AKI had the highest number of included studies for both specimens.

For all meta-analyses, the summary estimates showed the same positive direction of effect. Apart from some low patient number studies, or such with small event rates,^20^^,^^23^^,^^25^ the mean NGAL concentrations in urine (Fig 2) as well as in plasma (Fig 3) were increased for patients with AKI, patients with severe AKI, or patients needing RRT compared with patients without AKI or RRT, respectively.

For example, for plasma NGAL and patients with AKI (N=680) versus those without AKI (N=2,733), the mean NGAL concentration difference at admission was 86 (95% CI, 52-120) ng/mL, *P* < 0.001 (Fig. 3A). Although the mean plasma NGAL concentration difference of 258 patients with severe AKI versus 2704 without AKI was 151 (95% CI, 80-221) ng/mL, *P* < 0.001 (Fig. 3B).

In all meta-analyses, CS had the lowest mean NGAL concentration differences compared with the ICU or ED setting for each specimen type and all outcome measures. There was strong evidence for differences among settings in urine concentrations for AKI (*P* < 0.0001) and severe AKI (*P* < 0.0001) outcomes, but not for RRT (*P* = 0.78). However, the evidence for differences between settings for the mean plasma NGAL concentration was low for all outcome measures (*P* = 0.11 to *P* = 0.21). For plasma NGAL, the ICU setting showed the highest mean NGAL concentration difference in each setting. For urine NGAL, the largest study was performed in the ED,^13^ and showed the highest mean NGAL concentration difference for all outcome measures.

### Data Heterogeneity

The meta-analyses for AKI and severe AKI had considerable heterogeneity with I^2^ between 80% and 95%, and slightly lower for plasma NGAL than urine NGAL. For RRT, the observed heterogeneity was somewhat lower with I^2^ of 68.2% and 42.9% for urine and plasma, respectively. Regarding the subgroups for the clinical setting, heterogeneity was lower in CS than in ED or ICU settings.

### Subgroup Analyses

In summary, there were no differences or systematic funnel plot asymmetry between studies using and those studies not using the UOC for AKI classification for any of the outcome measures or either of the specimen types (Item S2.4).

### Post Hoc Analysis

#### Identification of Patients With AKI stage 1S

In Item S2.5, we calculated the difference of the individual studies’ mean NGAL concentration at admission and their NGAL concentration at the maximum Youden index derived from the area under the curve-ROC values to predict AKI.^6^ According to the formula ([Youden index concentration] − [mean NGAL concentration]), studies with a negative result included fractions of patients with higher NGAL concentrations in non-AKI patients than the respective threshold to predict sCr-based AKI using NGAL. These fractions changed, whether the study-individual NGAL cutoff concentrations or the meta-analyzed cutoff concentrations were used (Table S4).^6^^,^^7^^,^^39^

## Discussion

This ancillary meta-analysis used individual reassessed study data to calculate the mean NGAL concentration differences of patients with AKI, severe AKI, and those with the necessity of RRT initiation compared with those not developing AKI, severe AKI, or needing RRT, respectively, after admission to the ED, ICU, or after CS.

We found that patients who developed an adverse outcome of interest presented with higher admission NGAL concentrations for all outcome measures, regardless of specimen. However, this pattern was not consistently observed across all studies, and individual studies showed negative concentration differences for the individual outcome measures: specifically, in multiple studies, NGAL concentrations were elevated in patients without sCr concentration-based AKI. We consecutively investigated further on these results and demonstrated in a post hoc analysis how they may point toward the potential presence of AKI stage 1S^2^ at the time of patient admission.

Despite methodological adjustments to harmonize the data of the contributing studies, this meta-analysis revealed statistical heterogeneity, likely attributable to differences in clinical settings and original study protocols.

The previous study found lower meta-analyzed NGAL cutoff concentrations for rule-in or rule-out of adverse events in the studies using the UOC compared with those studies that did not,^6^ whereas the present meta-analysis found no subgroup difference in mean NGAL concentrations regarding the use of UOC. Most likely, this is because of the direct dependency of the NGAL cutoff concentration on UOC as the outcome measure. Likewise, the finding of incrementally higher mean NGAL concentration difference with increasing AKI severity might be connected to the finding of increasing cutoff concentrations with increasing AKI severity in the previous study.^6^ It is of interest that even when derived from the same cohort, different effect sizes, such as the summary area under the curve values calculated in the previous paper and the MD in NGAL concentrations reflecting the summary difference in average values between event and nonevent groups, may vary or diverge in heterogeneity, magnitude, and direction because they capture different statistical dimensions of biomarker performance—classification accuracy versus absolute concentration shift—each influenced by sample variability, distribution shape, and outcome definitions.^40^^,^^41^

NGAL is one of the most extensively investigated renal biomarkers; however, no previous study assessed the distribution and difference of NGAL concentrations at admission to the ED or ICU or after CS of those patients with and without adverse events on a meta-data level. Additionally, there is no literature available on admission NGAL concentrations in relation to a cutoff concentration to predict AKI.

It is well known that small studies included in meta-analyses tend to show more extreme effects, such as outlier tendency.^42^ In the present analysis, there were only a few outlier studies,^22^ and some smaller studies or those with low event numbers contributing inverse effects to the meta-analyses.^20^^,^^23^^,^^25^

As expected, this meta-analysis confirmed the hypothesis that NGAL concentrations at ED or ICU admission or after CS were elevated for patients with subsequent AKI, severe AKI, or acute RRT initiation compared to those without. These increases were more pronounced for severe AKI than for AKI of all stages, pointing toward a dose-response relationship for severe AKI over AKI of all stages versus non-AKI groups. However, analyses comparing AKI to severe AKI or RRT were not performed because dependent group comparisons would introduce double-counting and therefore distortion of the pooled effect size. Still, our findings acknowledged the usefulness of NGAL levels for clinical risk assessment, specifically in ED or ICU settings or at admission when no dynamics in SCr are presently available.

Although high heterogeneity may reduce the robustness of the pooled estimates, the assessment of fewer studies may also provide useful insights with careful interpretation. Moreover, the included studies were reassessed using uniform methodology, and they reported consistent effect sizes; thus, the clinical relevance of said heterogeneity may be misleading.^43^ However, none of the previous meta-analyses either assessed or compared NGAL concentrations at patient admission. Accordingly, the objective of the present study was to resolve scientific uncertainty. It did so by combining available evidence and showing that increased heterogeneity may, at least in part, be clinically explainable by differences in sample material, clinical setting,^44^ and procedural protocols. Reanalysis of study data on the individual-patient level offers meaningful advantages.^45^ Specifically, the harmonization of data and the inclusion of previously unpublished calculations in the present meta-analysis may have inherently reduced publication bias and even improved the robustness of our results.^46^

The present data may assist clinicians in better estimating the magnitude of AKI severity or the need for acute RRT upon receiving the NGAL test results for clinical evaluation in different settings. The results support the utility of NGAL in early kidney risk stratification in the ICU and the ED, enabling clinicians to make informed decisions based on NGAL screening. Additional data may be needed for urine NGAL in the CS setting, where perioperative sampling of NGAL is considered to improve renal risk assessment at patient admission.^47^^,^^48^ Of interest, although heterogeneity was low, the lowest MD in the urine NGAL concentration at admission was shown in the CS setting, challenging the urine NGAL applicability. This specific effect was not visible for plasma NGAL and might potentially be explained by supplementation of intravenous fluid, urine dilutional effects, and higher urine flow rates compared with patients in the ICU with impaired diuresis or urine concentration,^49^^,^^50^ but also because of the somewhat heterogeneous and well-prepared procedures of CS compared with critically ill patients and those admitted to the ED. Finally, subgroup analyses considering urine output to classify AKI revealed no difference for both specimens at patient admission.

In summary, the observed heterogeneity and outlined differences among settings highlight the need to interpret NGAL levels within the specific clinical setting and may also suggest that context-specific cutoff values or interpretation strategies may be necessary to improve accuracy and clinical utility.

The present analysis protocol focused on the inclusion of NGAL concentration measurements before AKI diagnosis or commencement of RRT. Of clinical interest, we identified multiple studies on urine or plasma NGAL levels with higher NGAL concentrations in the non-AKI group compared to reference intervals derived from healthy individuals.^51^ Post hoc analyses showed that these elevated NGAL concentrations at patient admission likely identify those with subclinical AKI.^2^ Such patients are at risk of progression to clinical AKI and need RRT initiation and are at increased risk of mortality compared with those without elevated NGAL concentrations.^39^^,^^52^ In the previous meta-analysis, we identified approximately 23.5% of patients with available individual-study data as NGAL-positive and SCr-based (RIFLE criteria) AKI-negative.^6^ Unfortunately, the data acquired did not allow for patient-specific outcome assessments. However, because of elevated NGAL concentrations in non-SCr-AKI groups (AKI stage 1S^2^), we hypothesize that the summarized mean NGAL difference at admission was lower than expected when considering SCr-based AKI only. Yet, no consensus threshold or definition of AKI stage 1S^2^ is available.^39^

Because the mean NGAL concentrations at admission differed from the individually determined threshold concentrations which we previously calculated for optimal renal risk prediction,^6^ we suggest that sequential measurements of NGAL may be of actionable use in the clinical course to refine renal assessment considering rule-in and rule-out algorithms.

As an ancillary study of a previous meta-analysis,^6^ the present study was restricted to the individual study data acquired for the primary analysis. Therefore, this study was limited to an adult population and did not include unpublished studies.^53^ However, the concept of individual study data reassessment enabled data harmonization and estimation of the MDs between groups with and without adverse events to be pooled directly in the prespecified meta-analyses. Not all authors who were initially requested to contribute their individual-study data participated or provided enough data to include in the present analysis. This analysis is limited by variations in study protocols; although RIFLE criteria were used for harmonization, not all studies incorporated urine output criteria. We refrained from additional adjustment for confounders such as sepsis or chronic kidney disease, as the number of studies and data was limited. Moreover, the primary study was not intended to provide sufficiently powered subgroup results but to estimate cross-sectional results potentially applicable in multidisciplinary or unclear clinical settings to facilitate renal risk assessment.

Finally, we acknowledge that the biological variability for NGAL and analytical assay and interassay variation all need to be considered when deriving clinical implications in reference to the MD calculated in the present study.^54^, ^55^, ^56^, ^57^

There seems to be a consistent association of elevated NGAL concentrations in patients who subsequently developed AKI or needed RRT over those who did not develop such adverse events. Our findings support the need for integrated risk models that, along with patients’ pre-existing conditions, simultaneously consider both NGAL and SCr concentration dynamics in relation to patient outcomes.^58^, ^59^, ^60^ Such studies might specifically assess the phenotyping, the differential indication, and the outcome of biomarker-triggered care bundles—such as those recommended by KDIGO—in patients with positive biomarker tests.^61^

## Conclusions

Notwithstanding the heterogeneity of findings and limitations inherent to this meta-analysis, our results demonstrated that admission NGAL levels differ between patients who subsequently developed AKI or required RRT and those who did not. Post hoc analyses indicated that multiple studies included patients with elevated mean NGAL concentrations without SCr-based AKI—potentially affected by AKI stage 1S^2^ at admission. The observed variability across studies and specimen types further underscores the importance of interpreting NGAL values within the specific clinical context and patient population.

## Supplementary Material

Supplementary File (PDF) Figures S1-S9; Items S1-S2,; Tables S1-S4.
