# Supplementary material for: Difference of Admission Neutrophil Gelatinase-Associated Lipocalin Concentration Between Patients Developing and Not Developing Acute Kidney Injury or Need for Acute Dialysis: An Ancillary Individual-Study Data Meta-Analysis (INDICATE–AKI)
Source: Kidney Med. 2026 Feb 2;8(5):101280. doi: 10.1016/j.xkme.2026.101280 (PMC13054045; doi:10.1016/j.xkme.2026.101280)
Supplement: Supplementary File (PDF) — Figures S1-S9; Items S1-S2,; Tables S1-S4. [file mmc1.pdf]

Difference of Admission Neutrophil Gelatinase-Associated Lipocalin Concentration Between Patients Developing and Not Developing Acute Kidney Injury or Need for Acute Dialysis: An Ancillary Individual-Study Data Meta-Analysis (INDICATE–AKI)

Supplementary Material

The NGAL Meta-Analysis Investigator Group

Annemarie Albert[1], Louisa Blume[2,3], Salvatore Di Somma[4], Mina Hur[5], Rinaldo Bellomo[6,7]†, Prasad Devarajan[8], Tobias Breidthardt[9], Fabrice Camou[10], Sidney Chocron[11], Dinna Cruz[12], Hilde RH. de Geus[13], Kent Doi[14], Zoltan H. Endre[15], Mercedes Garcia-Alvarez[16], Michael Haase[17,18], Anja Haase-Fielitz[19,20], Peter Buhl Hjortrup[21,22], Georgios Karaolanis[23], Cemil Kavalci[24], Hanah Kim[5], Sebastian Lange[1], Philipp Lauten[25,26], Paolo Lentini[27], Christoph Liebetrau[28,29], Miklós Lipcsey[30], Johan Mårtensson[31], Christian Müller[9], Serafim Nanas[32], Thomas L. Nickolas[33], John W. Pickering[34,35], Chrysoula Pipili[32], Claudio Ronco[36], Guillermo Rosa-Diez[37], Azrina Md Ralib[38], Karina Soto[39], Philipp Stieger[2], Antonia Zapf[40], Rüdiger C. Braun-Dullaeus[2], and Christian Albert[1,2]

Corresponding Author
Christian Albert
University Clinic for Cardiology and Angiology, Otto-von-Guericke University Magdeburg, Germany
Christian.Albert@med.ovgu.de
https://orcid.org/0000-0002-6956-9962

Study Registration
https://www.crd.york.ac.uk/prospero/display_record.php?ID=CRD42016042735

Complete author information is provided at the end of the document.

Supplemental Material Content

S1 Supplementary Methodology 3

S2 Supplementary Results 6

S2.1 Supplementary characteristics of included studies 6

S2.2 Assessment of publication bias and study selection-bias 8

S2.2.1 Funnel plot interpretation 11

S2.3 Quality Assessment 12

S2.4 Subgroup analyses for the use of urine output criterion for AKI 14

S2.4.1 Forest Plots 14

S2.4.2 Funnel Plots 20

2.5 Identification of patients with subclinical AKI (AKI stage 1S) 23

2.5.1 Difference of individual studies mean NGAL to the maximum Youden Index for AKI 23

S2.6 Mean NGAL concentrations according to RIFLE stages 26

Supplementary References 28

Abbreviations 29

Authors' affiliations 29

Supplementary Item 1. Supplementary Methodology

All studies included in the present analysis used CE-marked (Conformité Européenne) and certified widely available clinical laboratory platforms featuring superior turn-around times and reproducibility compared to enzyme-linked immuno-sorbent assay or research kits. However, potential interassay and test variability should be considered when interpreting results from the various platforms measuring NGAL, which may affect the transferability of results between assays.

Supplementary Table 1. Overview of included clinical laboratory platforms for measurement of NGAL

Test description	Sample material	Test type	Measure range	Manufacturer/licensing
TRIAGE NGAL TEST	Urine/Plasma	Point-of-Care immunoassay	15 – 1,300 ng/mL	Alere; Biosite Inc., San Diego, CA, USA/ QuidelOrtho Corporation, San Diego, CA, USA
ARCHITECT Analyzer	Urine	Chemiluminescent microparticle immunoassay	10 – 1,500 ng/mL*	Abbott, Abbott Diagnostics, Abbott Park, IL, USA
THE NGAL TEST†	Urine/Plasma	Particle-enhanced turbidimetric immunoassay	25 – 5,000 ng/mL	BioPorto Diagnostics A/S, Hellerup, Denmark; Bioporto, Needham, MA, USA

*Using an automated dilution procedure, the ARCHITECT can report values up to 6,000 ng/mL.

†Available on a variety of automated clinical chemistry analyzers; The ProNephro AKI™ (NGAL) test is cleared for risk assessment of pediatric AKI in the United States of America. The test run time is approximately 10 minutes.

All of the above NGAL tests are CE-marked (Conformité Européenne) and available for in-vitro diagnostic use in Europe.

Abbreviations: NGAL, neutrophil gelatinase-associated lipocalin

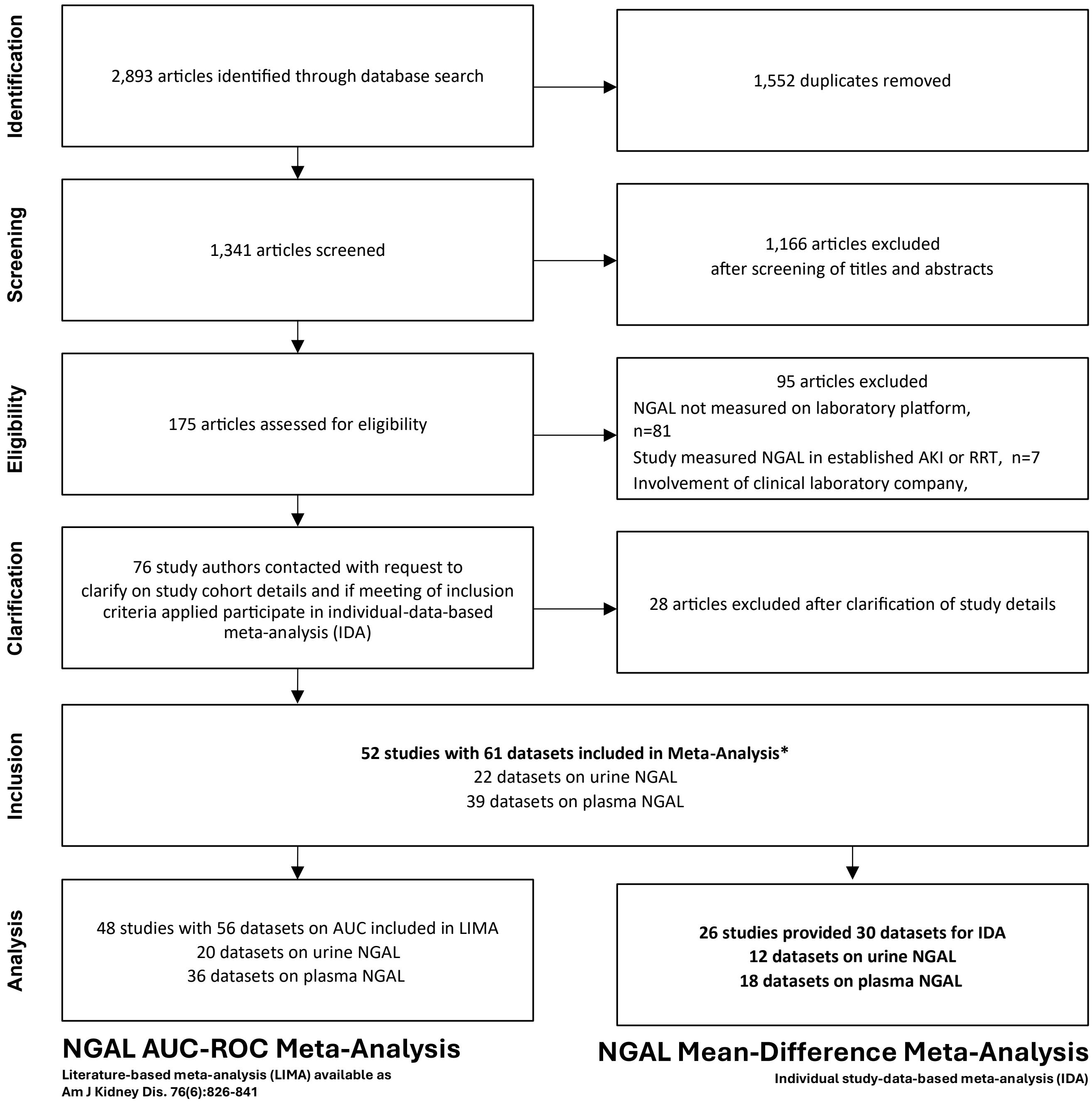

Figure S1. Study flow chart for INDICATE.

Note: The literature-based analysis (LIMA) of NGAL's discriminative AUC-ROC (area under the receiver operator characteristic curve) for the outcome measure AKI, severe AKI and acute RRT initiation is available in: Albert C. et al. Neutrophil Gelatinase-Associated Lipocalin Measured on Clinical Laboratory Platforms for the Prediction of Acute Kidney Injury and the Associated Need for Dialysis Therapy: A Systematic Review and Meta-analysis. ***Am J Kidney Dis***. 76(6):826-841. doi: 10.1053/j.ajkd.2020.05.015.[1]

Mean NGAL values respecting the inclusion requirements of the present individual study-data meta-analysis were not available in the literature.

Supplementary Table 2..Excerpt from the original request sheet for individual study data reassessment relevant to the present ancillary analysis. The complete data request sheet is available supplementary to the preceding publication.[1]

**Citation of publication:**	
**Sample size**	N=________
**Number of patients with**	RIFLE-AKI no: N=<br>RIFLE-AKI yes: N=<br>RIFLE R: N=<br>RIFLE I: N=<br>RIFLE F: N=<br><br>RRT yes: N=<br>RRT no: N=

**NGAL concentration, ng/mL**<br>(at ICU/ED admission)	No RIFLE: Mean ..., SD ...<br>RIFLE total: Mean ..., SD ...<br>RIFLE R: Mean ..., SD ...<br>RIFLE I: Mean ..., SD ...<br>RIFLE F: Mean ..., SD ...<br>RIFLE I+F: Mean ..., SD ...<br><br>No RRT: Mean ..., SD ...<br>RRT: Mean ..., SD ...

S2 Supplementary Results

S2.1 Supplementary characteristics of included studies

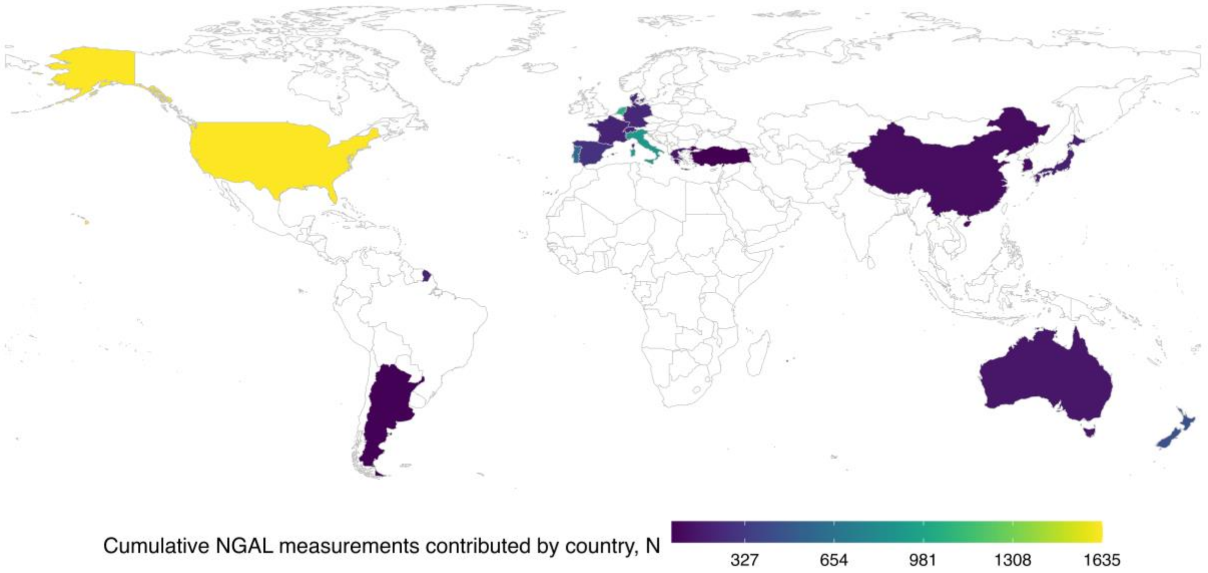

Supplementary Figure 2. World map illustrating the international and multicontinental data contribution to the meta-analysis. The heatmap illustrates the number of summary urinary and plasma neutrophil gelatinase-associated lipocalin (NGAL) measurements according to country. Total number of included NGAL measurements N=6655 from a total of 17 countries from 5 continents.

Supplementary Table 3. Data contribution and characteristics per study and country separated by specimen that NGAL was measured in.

Identifier	Citation	Country	Setting	Patient Number	AKI (female)	AKI (male)	Age AKI (SD)	Age No-AKI (SD)	SCr AKI (SD)	SCr No-AKI (SD)	SCr RRT (SD)	SCr No-RRT (SD)
Urine NGAL												
De Geus, 2011	Am J Respir Crit Care Med. 2011;183:907-14	The Netherlands	ICU	523	25	51	57.8 (17.5)	54.9 (17.3)	74.3 (21.2)	68.1 (17.7)	90.2 (21.2)	68.1 (18.6)
Nickolas, 2012	J Am Coll Cardiol. 2012;59:246-55	United States of America	ED	1635	132	313	68.3 (18.3)	62.9 (18.6)	90.2 (47.7)	86.7 (30.1)	143.2 (76.9)	86.7 (38.9)
Pipili, 2014	J Crit Care. 2014;29:692.e7-13	Greece	ICU	85	10	15	69.3 (15.7)	60.5 (20.0)	93.7 (41.6)	90.2 (63.7)	112.3 (79.6)	79.6 (35.4)
Liebetrau, 2013	Scand J Clin Lab Invest. 2013;73:392-9	Germany	CS	141	14	27	73.6 (8.6)	68.5 (10.8)	90.4 (26.7)	81.9 (34.8)	165.2 (72.0)	82.6 (29.6)
Hjortrup, 2015	Acta Anaesthesiol Scand. 2015;59:25-34	Denmark	ICU	100	11	15	66.9 (13.0)	63.5 (13.9)	74.7 (24.7)	87.6 (51.2)	100.1 (39.3)	83.0 (46.5)
Karaolanis, 2015	Hellenic J Cardiol. 2015;56:160-8	Greece	CS	69	4	9	72.9 (8.1)	64.1 (7.3)	98.1 (14.1)	81.3 (26.5)		
Mårtensson, 2015	Minerva Anestesiol. 2015;81:1192-200	Australia	ICU	35	2	4	70.8 (6.4)	61.1 (17.4)	99.4 (61.6)	88.1 (32.1)	63.9	90.8 (38.0)
Dai, 2015	Crit Care. 2015;6;19:223	People's Republic of China	ICU	112	26	26	49.5 (15.6)	52.2 (15.3)	80.2 (31.9)	76.8 (29.4)		78.4 (30.5)
Ralib, 2014	Crit Care. 2014;18:601	New Zealand	ICU	28	0	2	75.7 (0.1)	61.4 (16.8)	104.5 (7.8)	103.5 (30.0)	104.5 (7.8)	103.5 (30.0)
Varela, 2015	Ren Fail. 2015;37:327-31	Argentina	CS	66	5	10	73.0 (8.0	67.0 (11.0)	97.3 (24.8)	84.0 (20.3)	74.3 (3.5)	88.4 (22.1)
Garcia-Alvarez, 2015	J Cardiothorac Vasc Anesth. 2015;29:1480-8	Spain	CS	288	53	74	72.5 (9.5)	65.4 (12.6)	89.2 (31.7)	86.5 (34.6)	114.9 (51.6)	85.4 (30.3)
Haase, 2013	PLoS Med. 2013;10:e1001426	Germany	CS	100	2	7	74.8 (6.6)	64.2 (12.5)	101.9 (37.8)	92.9 (25.3)	125.4 (64.5)	92.1 (22.5)
Plasma NGAL												
De Geus, 2011	Am J Respir Crit Care Med. 2011;183:907-14	The Netherlands	ICU	523	25	51	57.8 (17. 5)	54.9 (17.3)	74.3 (21.2)	68.1 (17.7)	90.2 (21.2)	68.1 (18.6)
Cruz, 2010	Intensive Care Med. 2010;36:444-51	Italy	ICU	211	9	34	59.0 (19.0)	53.0 (20.0)	82.2 (22.1)	82.2 (33.6)	79.6	82.2 (31.8)
Di Somma, 2013	Crit Care. 2013 Feb 12;17:R29	Italy	ED	582	11	15	77.6 (11.8)	73.9 (13.5)	118.5 (69.0)	102.6 (44.2)		
Kim, 2013	Clin Biochem. 2013;46:1414-8	Republic of Korea	ICU	36	15	14	66.9 (17.3)	58.3 (17.0)	61.0 (31.8)	41.6 (12.4)	57.5	57.5 (30.1)
Park, 2015	Clin Biochem. 2015;48:44-9	Republic of Korea	CS	77	22	26	47.4 (14.4)	47.8 (12.1)	53.1 (13.3)	59.2 (8.8)		54.8 (12.4)
Perrotti, 2015	Ann Thorac Surg. 2015;99:864-9	France	CS	166	26	26	78.0 (5.0)	77.0 (6.0)	117.0 (36.0)	121.0 (47.0)	166.0 (32.0)	119.0 (43.0)
Doi, 2013	Crit Care. 2013,17:R270	Japan	CS	136	14	11	67.9 (11.0)	67.5 (11.3)	103.6 (49.7)	84.5 (34.3)	141.5 (51.3)	85.8 (35.4)
Camou, 2013	Ann Fr Anesth Reanim. 2013;32:157-64	France	ICU	50	11	29	61.4 (12.7)	56.0 (16.0)				
Breidthardt, 2012	Crit Care. 2012;16:R2	Switzerland	ED	207	12	5	81.9 (7.3)	77.7 (10.9)	137.3 (94.3)	119.0 (58.2)		
Hjortrup, 2015	Acta Anaesthesiol Scand. 2015;59:25-34	Denmark	ICU	124	16	16	65.9 (14.4)	65.0 (13.4)	77.9 (30.4)	88.9 (52.3)	111.9 (38.0)	82.1 (47.8)
Lipcsey, 2014	Biomarkers. 2014;19:22-8	Australia	CS	83	9	18	65.0 (10.0)	71.0 (10.0)	66.3 (18.6)	75.2 (25.6)	68.1 (13.3)	77.8 (24.8)
Ralib, 2014	Crit Care. 2014;18:601	New Zealand	ICU	40	2	3	59.7 (22.0)	61.8 (16.7)	99.0 (21.5)	102.0 (28.7)	160.9 (7.8)	101.5 (28.5)
Kavalci, 2014	J Pak Med Assoc. 2014;64:739-42	Türkiye	ED	60	17	8	69.9 (12.9)	66.4 (16.8)				
Mårtensson, 2015	Minerva Anestesiol. 2015;81:1192-200	Australia	ICU	35	2	4	70.8 (6.4)	61.1 (17.4)	99.4 (61.6)	88.1 (32.1)	63.9	90.8 (38.0)
Lentini, 2012	Crit Care Res Pract. 2012;2012:856401	Italy	ICU	98	23	15	62.3 (16.5)	63.0 (15.8)				
Katagiri, 2013	J Crit Care. 2013;28:564-70	Japan	ICU	65	7	8	65.1 (17.0)	60.2 (16.2)	91.1 (88.4)	65.4 (16.8)		70.7 (45.1)
Soto, 2013	Clin J Am Soc Nephrol. 2013;8:2053-63	Portugal	ED	602	74	109	62.1 (15.5)	57.5 (15.6)	83.1 (34.5)	68.1 (20.3)	114.9 (42.4)	71.6 (23.9)
Pickering, 2013	Blood Purif. 2013;35:295-302	New Zealand	ICU	378	7	11	65.6 (13.1)	59.4 (18.0)	93.4 (54.6)	80.7 (29.1)	130.0 (62.8)	79.6 (27.6)

Abbreviations: AKI, acute kidney injury (RIFLE classification); ICU, intensive care unit; ED, emergency department; CS, cardiac surgery; UOC, urine output criterion for AKI classification; RRT, renal replacement therapy; RIFLE, Risk, Injury, Failure, End-stage renal disease classification (Bellomo 2004); SCr, baseline serum creatinine (at patient admission/before surgery); SD, standard deviation

S2.2 Assessment of publication bias and study selection-bias

Supplementary Figure 3 a–c: Funnel plots for all performed urine neutrophil gelatinase-associated lipocalin meta-analyses. Subgroups for settings are highlighted as: Blue, Intensive Care Unit; Red, Cardiac Surgery; Green, Emergency Department. Studies with N≤1 event are left out as no standard error could be calculated. An in-detail discussion of the results is provided in S2.2.1.

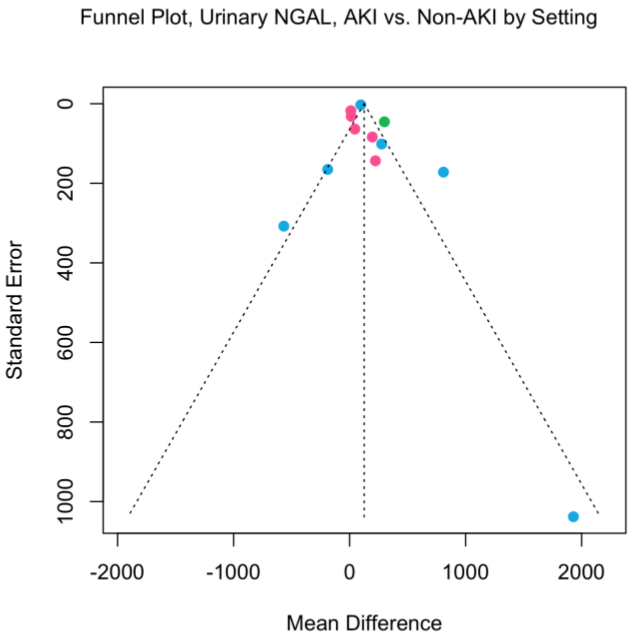

Supplementary Figure 3a: Funnel Plot of the Mean Difference measure as effect size versus the Standard Error for the comparison of RIFLE AKI versus Non-AKI patients using urine NGAL.

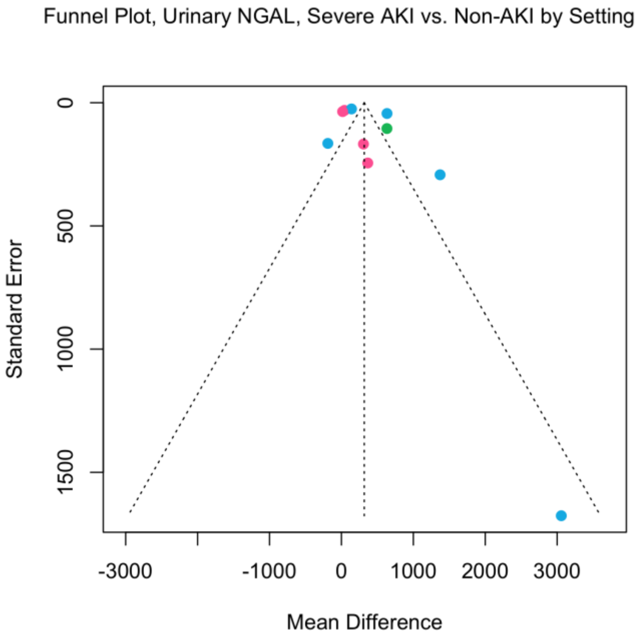

Supplementary Figure 3b: Funnel Plot of the Mean Difference measure as effect size versus the Standard Error for the comparison of Severe RIFLE AKI – defined as stages "Injury" or "Failure" – versus Non-AKI patients using urine NGAL.

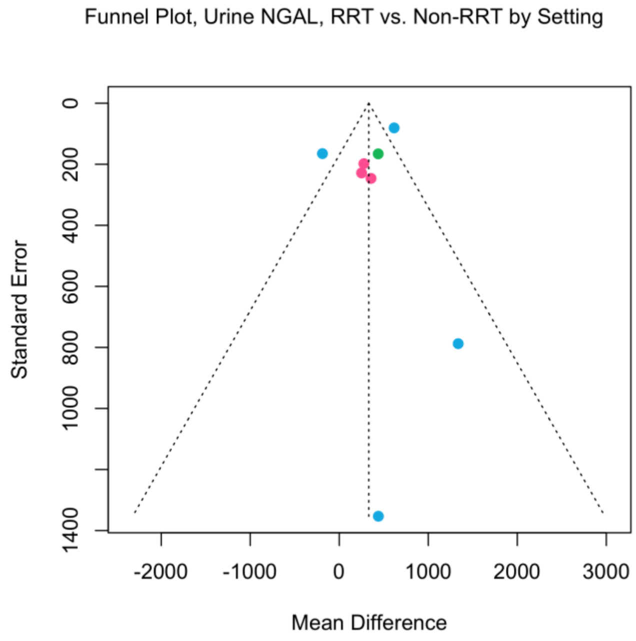

Supplementary Figure 3c: Funnel Plot of the Mean Difference measure as effect size versus the Standard Error for the comparison of RRT versus Non-RRT patients using urine NGAL.

Supplementary Figure 4 a–c: Funnel plots for all performed plasma neutrophil gelatinase-associated lipocalin meta-analyses. Subgroups for settings are highlighted as: Blue, Intensive Care Unit; Red, Cardiac Surgery; Green, Emergency Department. Studies with N≤1 event are left out as no standard error could be calculated. An in-detail discussion of the results is provided in S2.2.1.

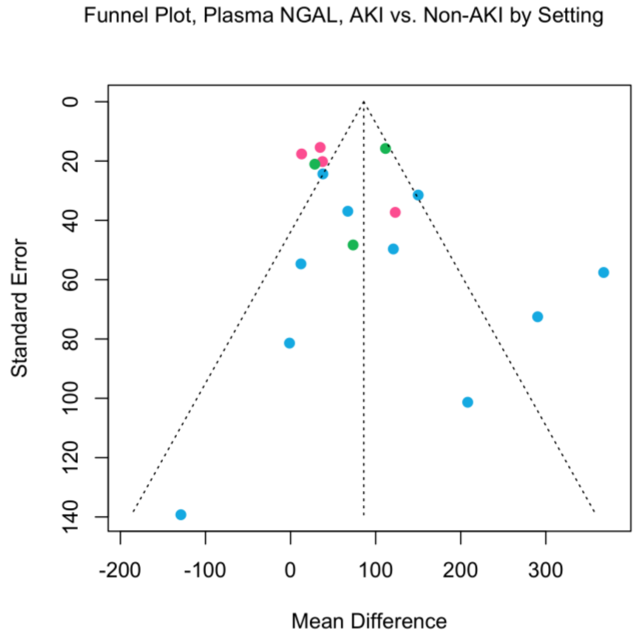

Supplementary Figure 4a: Funnel Plot of the Mean Difference measure as effect size versus the Standard Error for the comparison of RIFLE AKI versus Non-AKI patients using plasma NGAL.

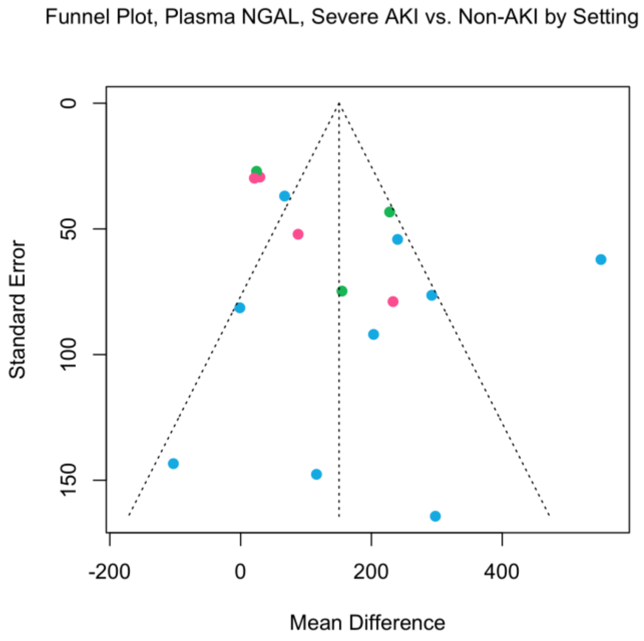

Supplementary Figure 4b: Funnel Plot of the Mean Difference measure as effect size versus the Standard Error for the comparison of Severe RIFLE AKI – defined as stages "Injury" or "Failure" – versus Non-AKI patients using plasma NGAL.

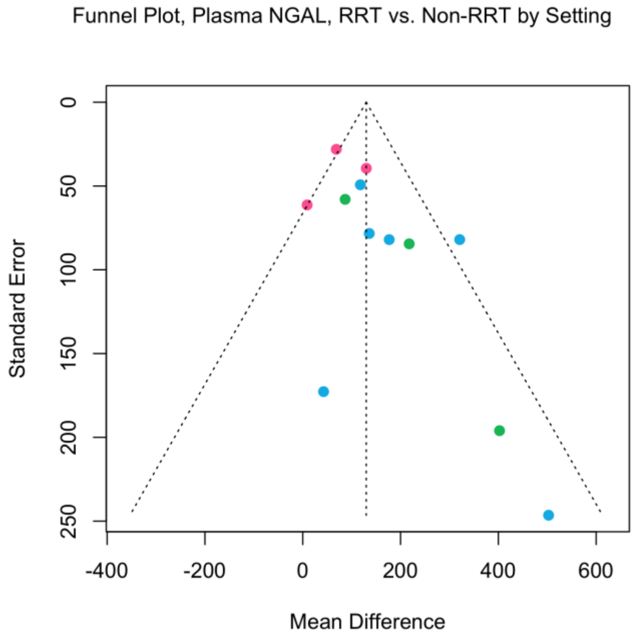

Supplementary Figure 4c: Funnel Plot of the Mean Difference measure as effect size versus the Standard Error for the comparison of RRT versus Non-RRT patients using plasma NGAL.

S2.2.1 Funnel plot interpretation

The funnel plots for the present data provided in **Figure S2a–c** and **S3a–c** do not exhibit obvious asymmetry, and hence provide no evidence for systematic selection bias or indication of small-study effects.[2] We note that the ability of funnel plots to detect such bias is limited when the number of studies is small[3] or heterogeneity between studies is present.[4] However, the present analysis is based on individual reassessed data on patient level according to prespecified uniform criteria enabling data harmonization. This is of relevance, as these specific data have not been published previously, and might therefore act as a corrective for potential funnel asymmetry.[5]

In addition to the limitations of funnel plots discussed in the main manuscript, the funnel plots must be interpreted under the condition, that they are representative of the secondary study aim of a previous meta-analysis. The primary study aim was to calculate cutoff values for NGAL derived from the area under the receiver operator characteristic (AUC) curve's sensitivity and specificity pairs to predict the development of AKI, severe AKI or necessity for RRT with 95% sensitivity, optimal combination of sensitivity and specificity and 95% specificity to facilitate renal risk prediction to rule-out or rule-in patients at risk. Thus, corresponding funnel plots used AUC and SE as metric in contrast to the mean difference and SE used in the present analysis.

Accordingly, hypothetically, studies from authors providing reanalyzed individual-study-data of their investigations may potentially be those with more favorable results for AUC–and derived cutoff values–as the primary study aim of the original publication. In the original investigation, however, in the results of subgroup analysis for studies providing individual study data and those studies analyzed from the literature, only, we did not find pooled area under the receiver operator characteristic curve values favoring individual over literature analyses or vice versa, where specifically systematic tendency towards studies with higher AUCs potentially more likely providing individual-study data would indicate selection bias.

Both individual-data based meta-analyses did not include unpublished studies as such did not undergo peer-review. However, the genuine setting of the present meta-analysis is the assessment of individual reassessed data on patient level according to prespecified uniform criteria. Accordingly, this data was not published previously. This might also affect symmetry of the funnel plots and correct potential asymmetry. We therefore refrained from application of funnel-plot asymmetry tests.

In summary, as also for the present secondary study aim of comparing NGAL concentrations in those patients developing and those not developing adverse events as outcome measure, together with results from the primary analysis, did not indicate any systematic funnel asymmetry–or regarding the original meta-analysis– systematic difference between the individual data and literature-based meta-analysis, the assessment gave no indication of relevant publication bias.

The corresponding analyses regarding risk of bias referred to in the above paragraph are available supplementary to the preceding publication.[1]

S2.3 Quality Assessment

For quality assessment Review Manager (RevMan, version 5.3, Copenhagen: The Nordic Cochrane Centre, The Cochrane Collaboration, 2014) was used. The quality assessment regarding risk of bias and applicability concerns were assessed previously for the primary study aim regarding the ability of NGAL to predict AKI or RRT necessity using the QUADAS-2 (Quality Assessment Tool for Diagnostic Accuracy, version 2) tool available in RevMan. The results are therefore also applicable for the present assessment. The quality of studies providing reassessed individual study data was evaluated before (**Supplementary Figure 4a**) and after application of prespecified harmonization criteria (**Supplementary Figure 4b)**. For the literature-based assessment, risk of bias and applicability was moderate. After data harmonization, Quadas-2 showed improvement for risk of bias of the index test. Further interpretation of the quality analysis is available supplementary to the to the preceding publication.[1]

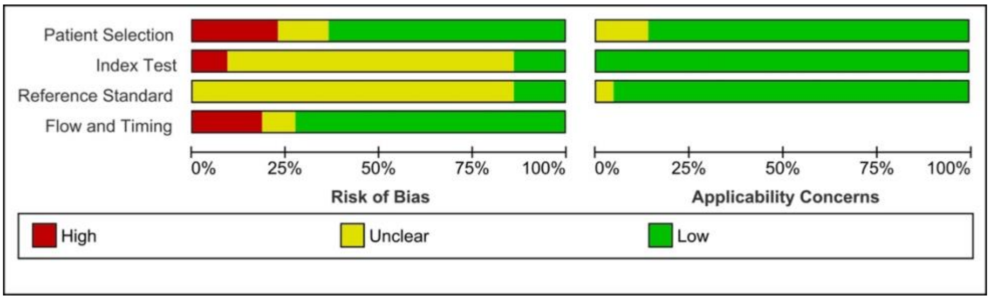

Supplementary Figure 5a: Overview about the risk of bias and applicability concerns using the QUADAS-2 tool based on the complete literature-based analysis

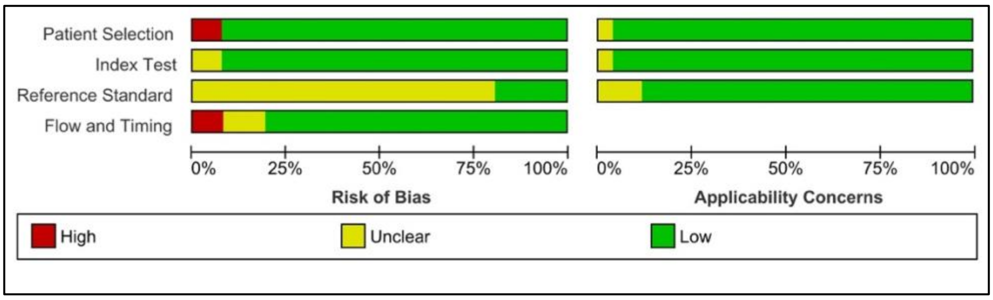

Supplementary Figure 5b: Overview about the risk of bias and applicability concerns using the QUADAS-2 tool based on the individual-data analysis after harmonization of data according to standardized AKI definitions (RIFLE), timing of NGAL sampling and corresponding prespecified patient inclusion criteria.

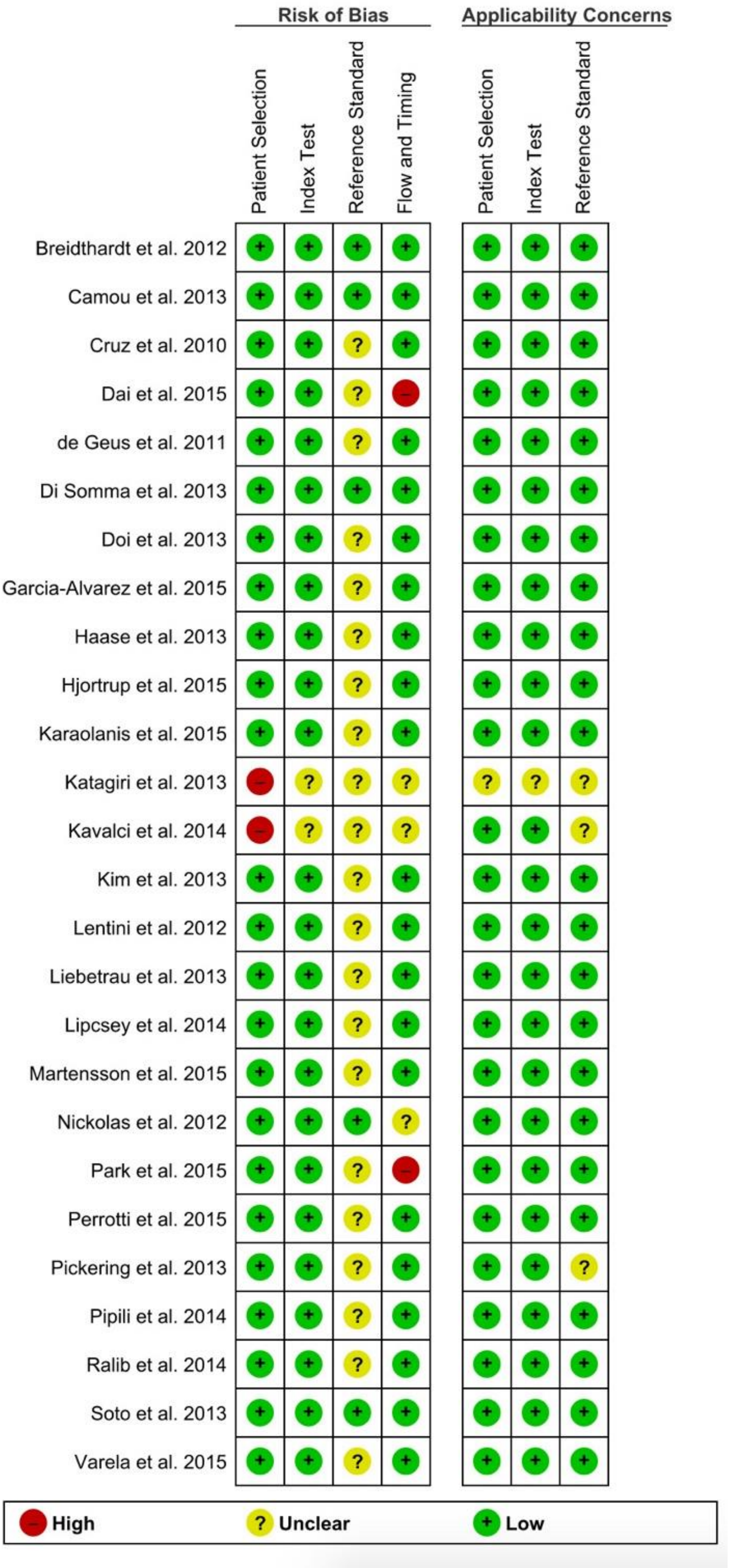

Supplementary Figure 5c: Risk of bias and applicability concerns using the QUADAS-2 tool for each included study based on the individual-data analysis after harmonization.

S2.4 Subgroup analyses for the use of urine output criterion for AKI

The Urine output criteria for the RIFLE stages are defined as follows:
Risk: <0.5 mL/kg/h × 6 h; Injury: <0.5 mL/kg/h × 12 h; Failure: <0.3 mL/kg/h × 24 h or anuria × 12 h.
(Bellomo R *et al.* ***Crit Care***. 2004;8:R204. doi: 10.1186/cc2872)[6]
The non-use of the urine output criterion for the classification of AKI may potentially confound the allocation of individual patients to be diagnosed to have AKI and their individual allocation to AKI stages, respectively. Consecutively, the methodology and allocation of patients to have AKI may differ between included studies.
In chapter S2.4.1 meta-analyses for the assessment of group differences between those studies using and those not using the urine output criterion of the RIFLE classification for staging of AKI are performed. In this effort all studies are regrouped according to this setting in the meta-analysis algorithm and illustrated in the following forest plots. While the test for overall effect will provide the same results as in the main manuscript, the relevant results of this chapters' analyses are the within group differences assessed by χ^2–test, where a significant test result would indicate relevant subgroup differences. In the following figures we provide results on the hypothesis that the use of urine output criterion may influence the mean difference of NGAL measured in either urine or plasma between patients with or without the outcome measure AKI, severe AKI and acute RRT initiation, respectively.

S2.4.1 Forest Plots

Study	AKI Total	AKI Mean	AKI SD	Non-AKI Total	Non-AKI Mean	Non-AKI SD	MD	95%-CI	Weight
**Setting = Consideration of Urine Output Criterion**									
De Geus, 2011	76	1080.00	1477.00	447	271.00	625.00	809.00	[471.92; 1146.08]	3.3%
Pipili, 2014	25	576.97	478.64	60	300.65	257.83	276.32	[77.68; 474.96]	6.9%
Liebetrau, 2013	41	48.24	82.74	100	37.73	120.52	10.51	[-24.12; 45.14]	16.3%
Mårtensson, 2015	6	182.70	300.30	29	750.00	1520.00	-567.30	[-1170.44; 35.84]	1.2%
Dai, 2015	52	243.27	19.03	60	147.03	10.12	96.24	[90.47; 102.01]	17.0%
Varela, 2015	15	112.00	232.00	51	67.20	156.80	44.80	[-80.24; 169.84]	10.8%
Haase, 2013	9	288.07	427.52	91	65.33	159.04	222.73	[-58.48; 503.95]	4.4%
**Random effects model**	**224**			**838**			**115.71**	**[32.30; 199.13]**	**59.8%**
Heterogeneity: I^2 = 87.9%, τ^2 = 6135.8458, p < 0.0001									
**Setting = No Consideration of Urine Output Criterion**									
Nickolas, 2012	445	399.02	944.48	1190	98.96	269.06	300.06	[210.99; 389.13]	13.2%
Hjortrup, 2015	26	2812.00	5129.00	74	883.10	2208.40	1928.90	[-105.78; 3963.58]	0.1%
Karaolanis, 2015	13	45.00	105.90	56	31.30	92.90	13.70	[-48.80; 76.20]	14.9%
Ralib, 2014	2	144.15	182.22	26	333.14	526.49	-188.99	[-512.61; 134.63]	3.5%
Garcia-Alvarez, 2015	127	390.04	836.80	161	194.40	490.18	195.64	[31.59; 359.70]	8.6%
**Random effects model**	**613**			**1507**			**123.99**	**[-71.94; 319.92]**	**40.2%**
Heterogeneity: I^2 = 88.2%, τ^2 = 33131.6361, p < 0.0001									
**Random effects model**	**837**			**2345**			**125.43**	**[57.33; 193.54]**	**100.0%**

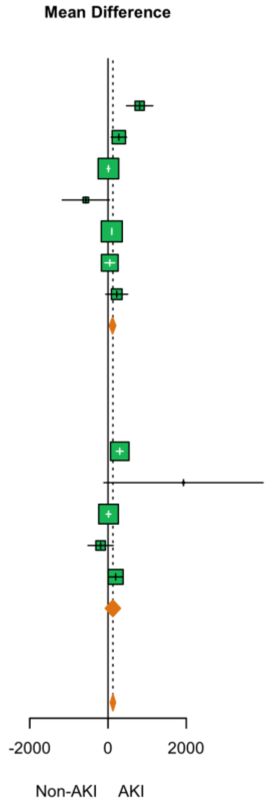

Heterogeneity: I^2 = 86.9%, τ^2 = 7110.9724, p < 0.0001
Test for subgroup differences: χ^2_1 = 0.01, df = 1 (p = 0.9392)
Chi-Square = 83.86, P = < 0.0001, Degrees of Freedom = 11
MD = 125.43, 95% CI = [57.33; 193.54], Test for overall effect: Z = 3.61, P = 0.0003

Supplementary Figure 6a: Forest Plot for the mean difference of urine NGAL concentrations and standard deviation at admission for outcome measure AKI grouped by consideration of urine output criterion for AKI classification. There were no subgroup differences (χ^2–test P=0.939) between studies using and those studies not using the urine output criterion of the RIFLE classification for classification of AKI.

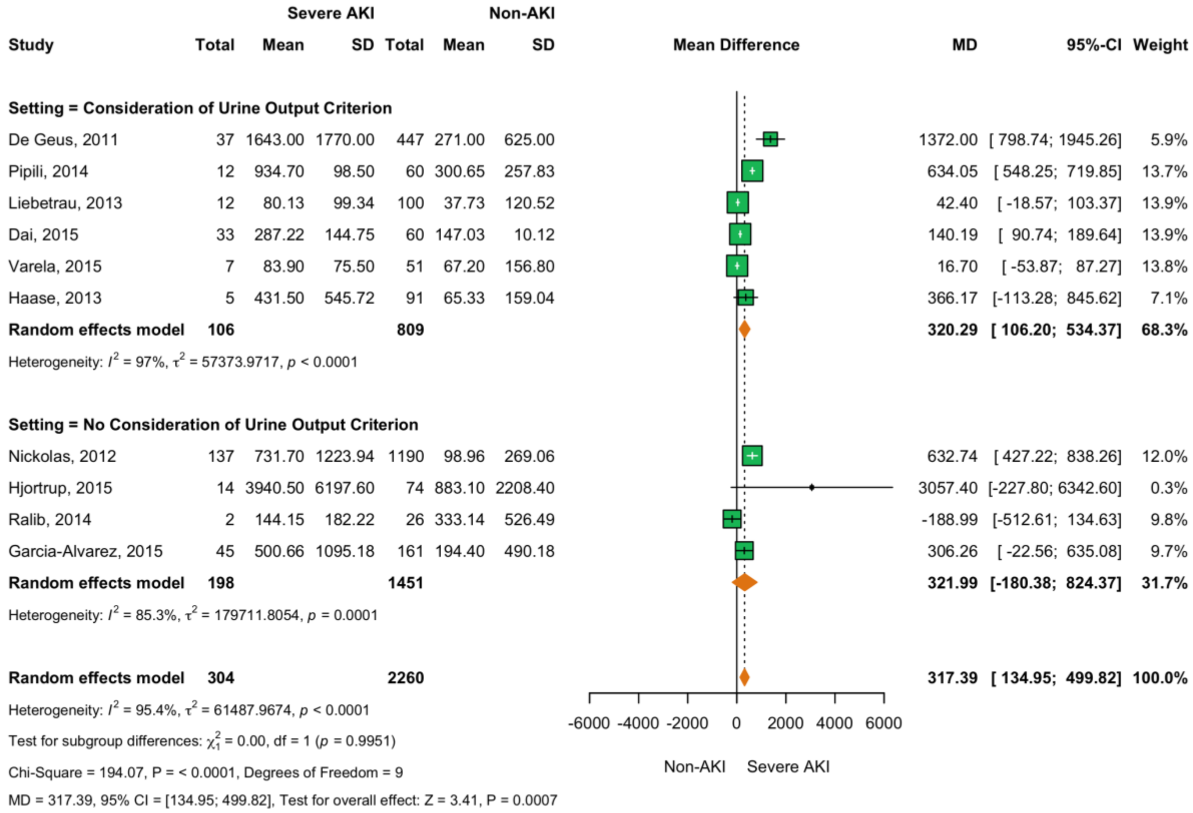

Supplementary Figure 6b: Forest Plot for the mean difference of urine NGAL concentrations and standard deviation at admission for outcome measure severe AKI grouped by consideration of urine output criterion for AKI classification. There were no subgroup differences (χ^2–test P=0.995) between studies using and those studies not using the urine output criterion of the RIFLE classification for staging of AKI.

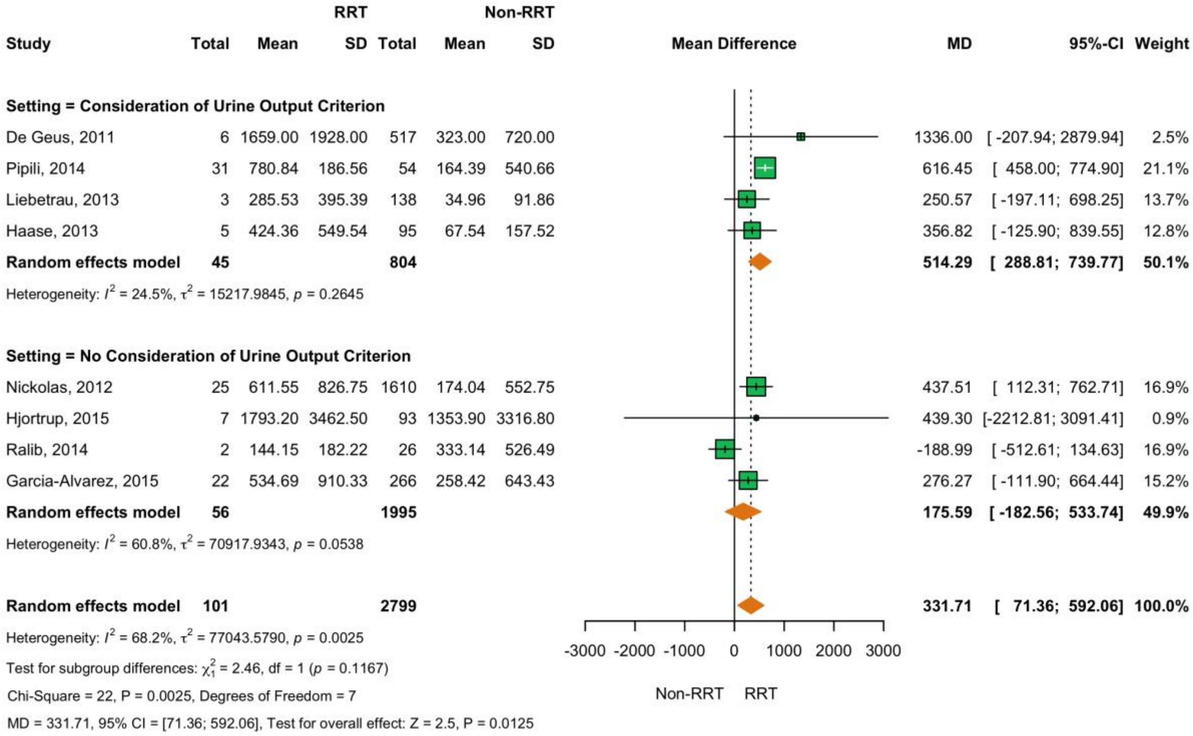

Supplementary Figure 6c: Forest Plot for the mean difference of urine NGAL concentrations and standard deviation at admission for outcome measure RRT grouped by consideration of urine output criterion for AKI classification. There were no subgroup differences (χ^2–test P=0.117) between studies using and those studies not using the urine output criterion of the RIFLE classification for staging of AKI.

Study	AKI Total	AKI Mean	AKI SD	Non-AKI Total	Non-AKI Mean	Non-AKI SD	MD	95%-CI	Weight
**Setting = Consideration of Urine Output Criterion**									
De Geus, 2011	76	329.00	268.00	447	179.00	143.00	150.00	[88.31; 211.69]	7.1%
Cruz, 2010	43	170.00	142.00	168	132.00	144.00	38.00	[-9.70; 85.70]	7.8%
Di Somma, 2013	26	206.30	244.60	556	132.80	133.00	73.50	[-21.17; 168.17]	5.4%
Camou, 2013	40	481.80	269.80	10	191.40	185.40	290.40	[148.29; 432.51]	3.6%
Mårtensson, 2015	6	149.00	120.50	29	136.80	128.50	12.20	[-94.96; 119.36]	4.8%
Pickering, 2013	18	144.44	153.04	360	77.16	150.14	67.28	[-5.10; 139.66]	6.5%
**Random effects model**	**209**			**1570**			**94.24**	**[33.01; 155.47]**	**35.2%**
Heterogeneity: I^2 = 72.2%, τ^2 = 3939.3017, p = 0.0029									
**Setting = No Consideration of Urine Output Criterion**									
Kim, 2013	29	258.20	188.60	7	387.10	356.60	-128.90	[-401.84; 144.04]	1.3%
Park, 2015	48	126.10	90.00	29	91.30	44.60	34.80	[4.60; 65.00]	8.6%
Perrotti, 2015	52	316.02	243.06	114	192.97	170.70	123.05	[49.93; 196.17]	6.5%
Doi, 2013	25	141.52	87.33	111	104.01	106.44	37.51	[-2.04; 77.06]	8.2%
Breidthardt, 2012	17	136.40	84.20	190	107.90	71.30	28.50	[-12.79; 69.79]	8.1%
Hjortrup, 2015	32	618.70	533.30	92	410.50	356.30	208.20	[9.60; 406.80]	2.3%
Lipcsey, 2014	27	129.00	80.00	56	116.00	64.00	13.00	[-21.52; 47.52]	8.4%
Ralib, 2014	5	206.75	160.77	35	207.97	225.67	-1.22	[-160.74; 158.30]	3.1%
Lentini, 2012	38	581.00	306.00	60	213.00	226.00	368.00	[255.15; 480.85]	4.6%
Katagiri, 2013	15	243.30	184.10	50	122.50	101.50	120.80	[23.48; 218.12]	5.3%
Soto, 2013	183	205.62	208.91	419	94.02	65.12	111.60	[80.70; 142.50]	8.5%
**Random effects model**	**471**			**1163**			**82.37**	**[39.03; 125.71]**	**64.8%**
Heterogeneity: I^2 = 83.7%, τ^2 = 3489.7933, p < 0.0001									
**Random effects model**	**680**			**2733**			**86.04**	**[51.74; 120.34]**	**100.0%**
Heterogeneity: I^2 = 80.3%, τ^2 = 3337.4954, p < 0.0001									
Test for subgroup differences: χ^2_1 = 0.10, df = 1 (p = 0.7565)									
Chi-Square = 81.22, P = < 0.0001, Degrees of Freedom = 16									
MD = 86.04, 95% CI = [51.74; 120.34], Test for overall effect: Z = 4.92, P = < 0.0001									

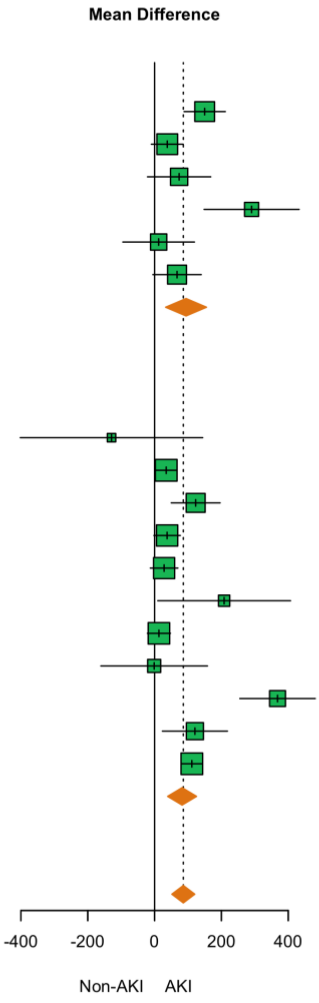

Supplementary Figure 6d: Forest Plot for the mean difference of plasma NGAL concentrations and standard deviation at admission for outcome measure AKI grouped by consideration of urine output criterion for AKI classification. There were no subgroup differences (χ^2–test P=0.757) between studies using and those studies not using the urine output criterion of the RIFLE classification for staging of AKI.

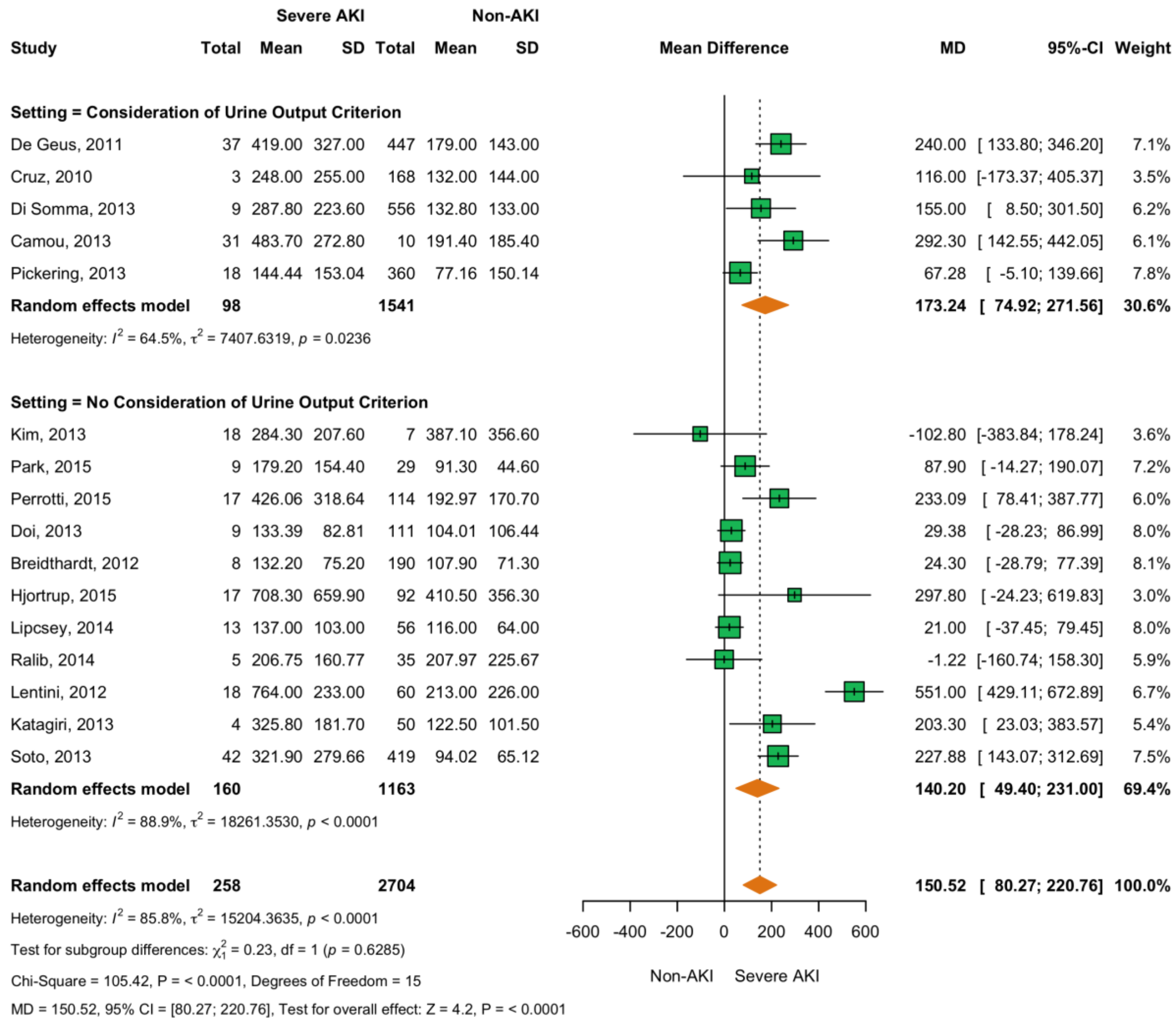

Supplementary Figure 6e: Forest Plot for the mean difference of plasma NGAL concentrations and standard deviation at admission for outcome measure severe AKI grouped by consideration of urine output criterion for AKI classification. There were no subgroup differences (χ^2–test P=0.629) between studies using and those studies not using the urine output criterion of the RIFLE classification for staging of AKI.

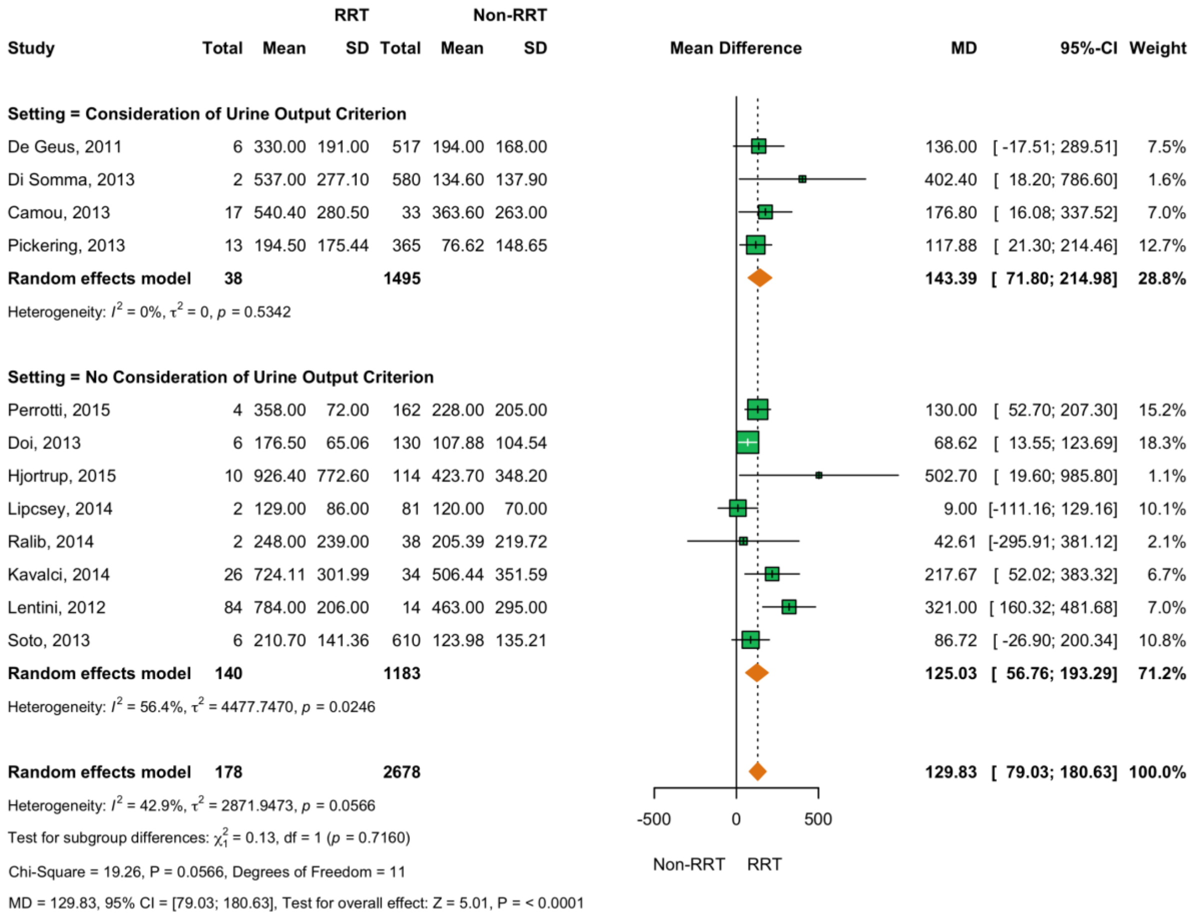

Supplementary Figure 6f: Forest Plot for the mean difference of plasma NGAL concentrations and standard deviation at admission for outcome measure RRT grouped by consideration of urine output criterion for AKI classification. There were no subgroup differences (χ^2–test P=0.716) between studies using and those studies not using the urine output criterion of the RIFLE classification for staging of AKI.

S2.4.2 Funnel Plots

In addition to the considerations discussed in **Supplementary chapter S2.2.1**, there was no obvious systematic asymmetry regarding the distribution of those studies considering (blue) versus those studies not considering (orange) the urine output criterion for AKI classification in the funnel plots. These findings indicate that there was no obvious influence on the mean difference and SE concerning publication bias and effect size of NGAL difference between the groups and outcome measures. Again, studies with N≤1 event were excluded from the respective meta-analysis as no standard error could be calculated.

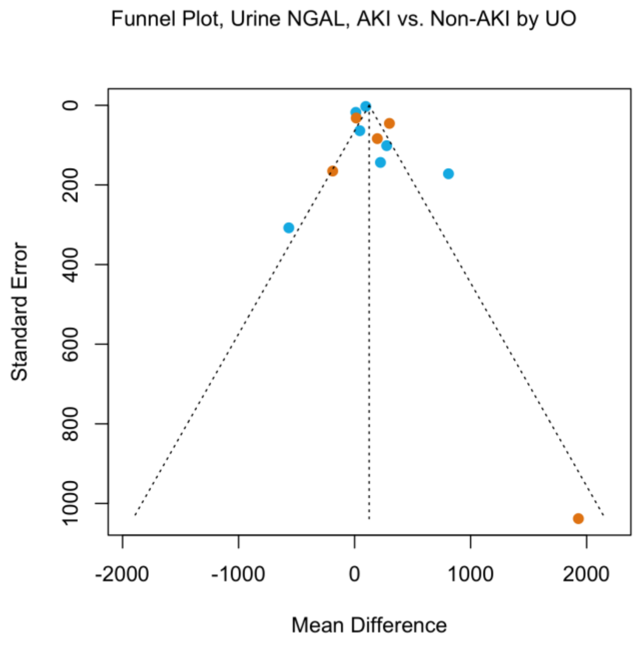

Supplementary Figure 7a: Funnel Plot of the mean difference measure as effect size versus the Standard Error for the comparison of RIFLE AKI versus Non-AKI patients using urine NGAL grouped by consideration of urine output criterion YES=blue, NO=orange.

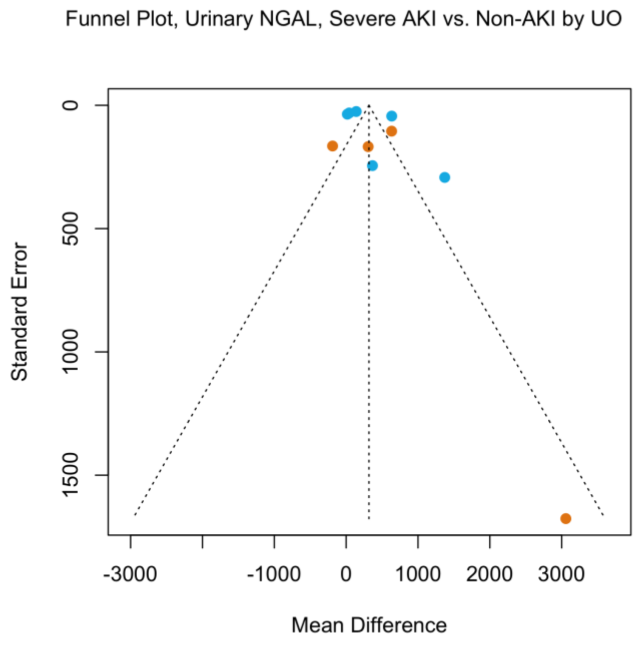

Supplementary Figure 7b: Funnel Plot of the mean difference measure as effect size versus the Standard Error for the comparison of Severe RIFLE AKI – defined as stages "Injury" or "Failure" – versus Non-AKI patients using urine NGAL grouped by consideration of urine output criterion YES=blue, NO=orange.

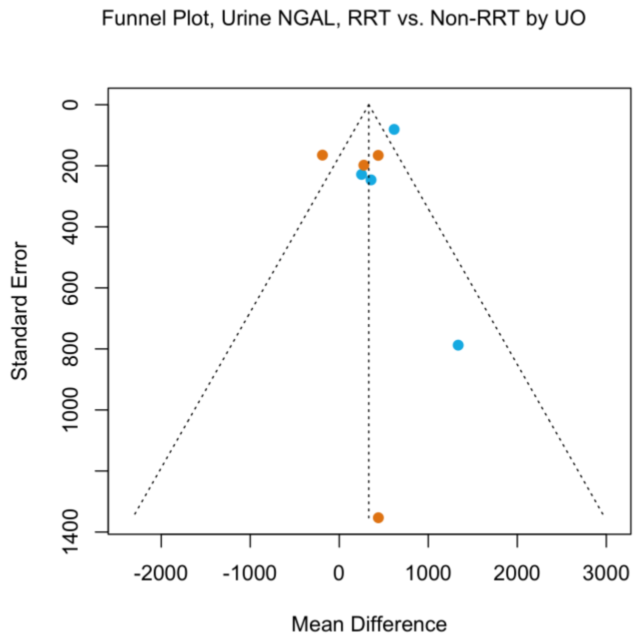

Supplementary Figure 7c: Funnel Plot of the mean difference measure as effect size versus the Standard Error for the comparison of RRT versus Non-RRT patients using urine NGAL grouped by consideration of urine output criterion YES=blue, NO=orange.

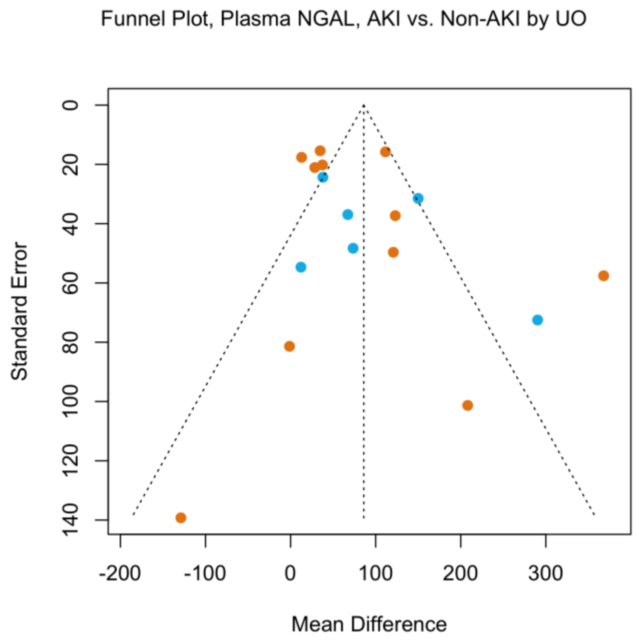

Supplementary Figure 7d: Funnel Plot of the mean difference measure as effect size versus the Standard Error for the comparison of RIFLE AKI versus Non-AKI patients using plasma NGAL grouped by consideration of urine output criterion YES=blue, NO=orange.

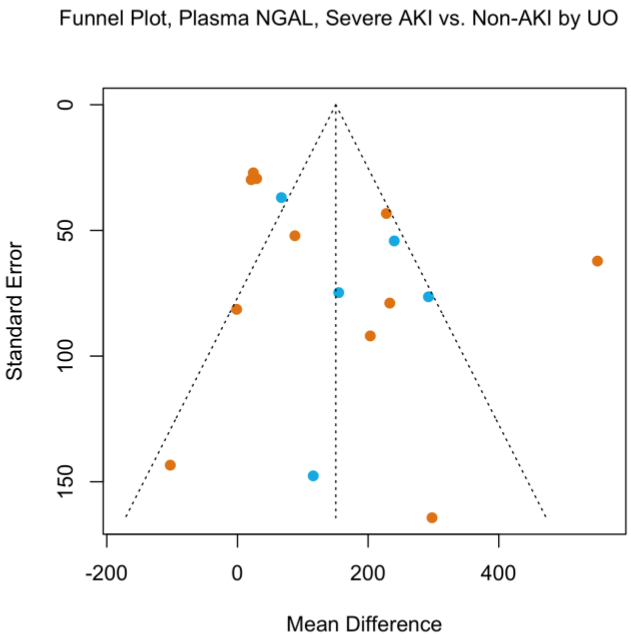

Supplementary Figure 7e: Funnel Plot of the mean difference measure as effect size versus the Standard Error for the comparison of Severe RIFLE AKI – defined as stages "Injury" or "Failure" – versus Non-AKI patients using plasma NGAL grouped by consideration of urine output criterion YES=blue, NO=orange.

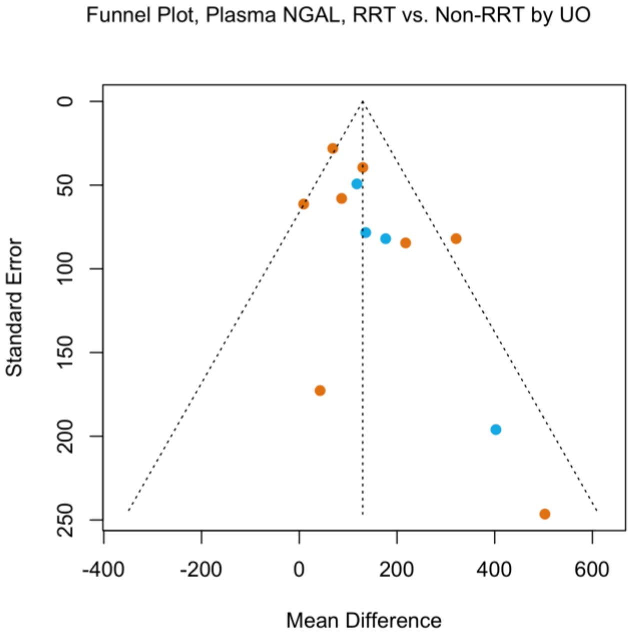

Supplementary Figure 7f: Funnel Plot of the mean difference measure as effect size versus the Standard Error for the comparison of RRT versus Non-RRT patients using plasma NGAL grouped by consideration of urine output criterion YES=blue, NO=orange.

2.5 Identification of patients with subclinical AKI (AKI stage 1S)

2.5.1 Difference of individual studies mean NGAL to the maximum Youden Index for AKI

We calculated the difference of the individual studies' mean NGAL concentration at admission to their individual NGAL concentration at the maximum Youden index derived from the AUC-ROC to predict AKI (see also **Supplementary Table 4** on page 24) using the formula: ([Youden Index]–[mean NGAL concentration]). Accordingly, studies with negative result (negative slope in Figure S7 a and b) included fractions of patients with higher NGAL concentrations in Non-AKI patients than the respective optimal threshold to predict SCr-based AKI by means of urine or plasma NGAL, respectively. Accordingly, if some patients (by raising the mean NGAL in Non-AKI group) had concentrations above the threshold to predict AKI may be diagnosed as having subclinical AKI or AKI stage 1S in reference to recommendations by Ostermann M *et al.*[7]
However, the data collected in this analysis do not allow any conclusions to be drawn about the patient-related outcome.

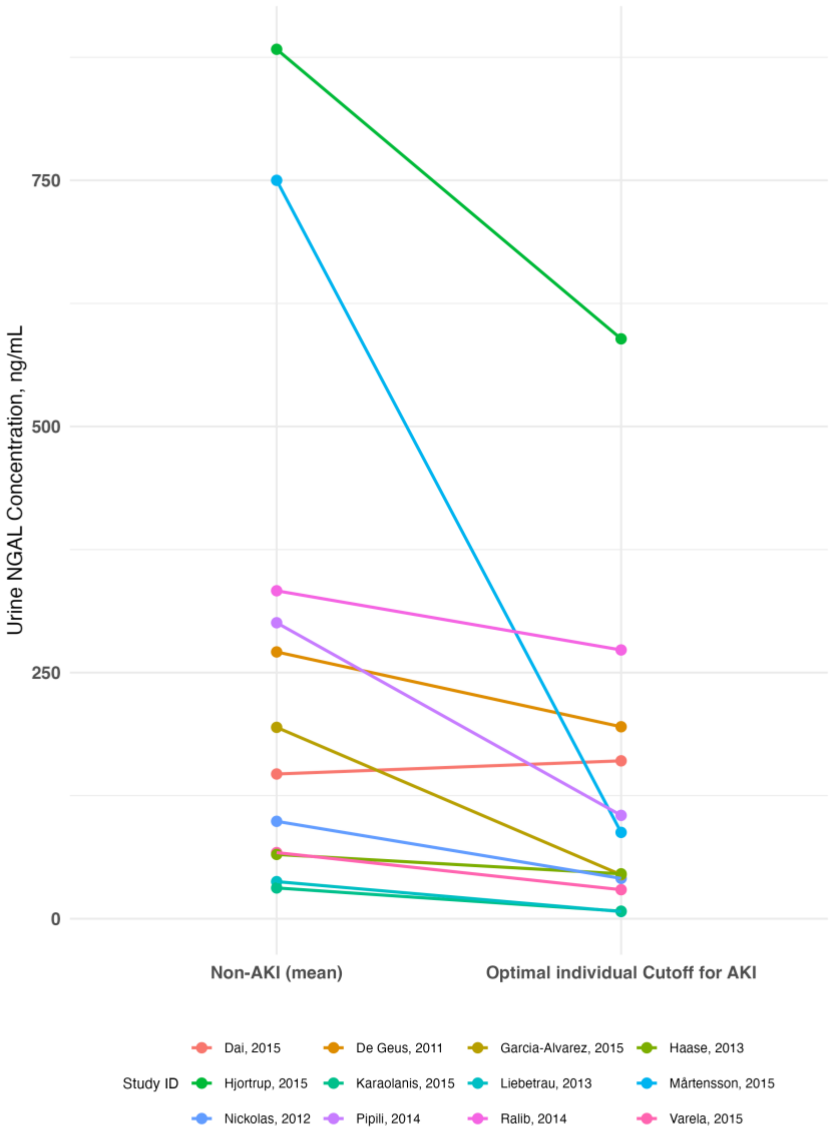

Supplementary Figure 8a: Difference of individual studies mean urine NGAL to their maximum Youden Index derived from the ROC-curve for AKI prediction.

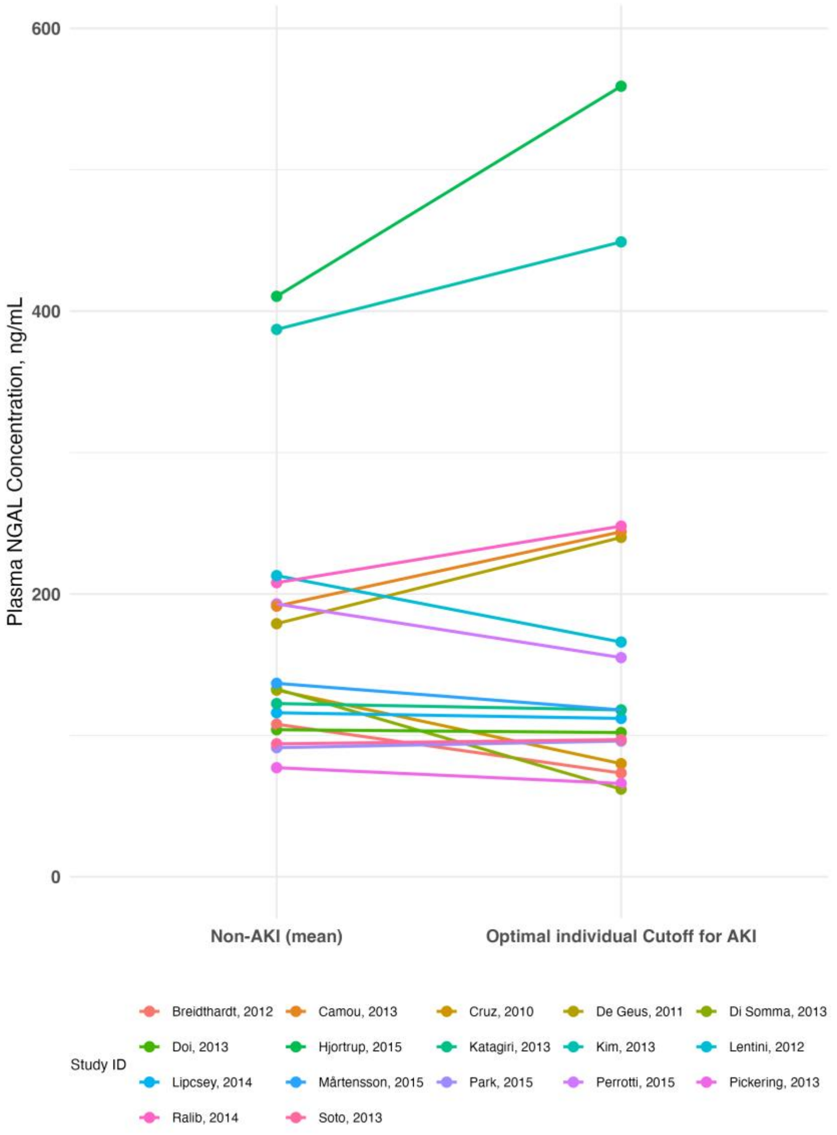

Supplementary Figure 8b: Difference of individual studies mean plasma NGAL to their maximum Youden Index derived from the ROC-curve for AKI prediction.

Still, results of the present meta-analysis point towards the presence of subclinical AKI (AKI stage 1S) following the definition proposed by Osterman M *et al*. (Jama Netw Open 2020;3:e2019209) when considering mean NGAL concentrations at admission to the ED or ICU, in relation to individual study-specific optimal cutoff values to predict AKI.

Subclinical acute kidney injury is particularly of clinical interest when identified at ED or ICU admission, as early identification of subclinical AKI may provide the time utility window for therapeutic implications. Timely initiation of kidney-protective treatments—such as recommended KDIGO care bundles—has been shown to improve outcomes for SCr-based AKI and composite measures like adverse kidney events.

Supplementary Table 4. Identification of studies with patients with subclinical AKI

Reference	max. Youden Index for AKI of all stages	Difference of Youden to mean NGAL for Non-AKI of each individual study	Difference to meta-analyzed Youden cutoff concentration for AKI*
Urine NGAL			
Mårtensson et al., 2015	87.7	-662.30	-669.00
Pipili et al., 2014	105	-195.65	-219.65
Hjortrup et al., 2015	589	-294.10	-802.10
de Geus et al., 2011	195	-76.00	-190.00
Dai et al., 2015	160.4	13.37	-66.03
Ralib et al., 2014	273	-60.14	-252.14
Nickolas et al. 2012	41	-57.96	-17.96
Liebetrau et al., 2013	7.3	-30.43	43.27
Karaolanis et al., 2015	7.7	-23.60	49.70
Varela et al., 2015	29.5	-37.70	13.80
Garcia-Alvarez et al., 2015	44.7	-149.70	-113.40
Haase et al., 2013	45.65	-19.68	15.67
Median (25–75 CI)	66.68 (38.13–169.10)	-59.05 (-161.19– -28.72)	-89.72 (-227.77–14.27)
Plasma NGAL			
Cruz et al., 2010	80	-52	33
Camou et al., 2013	244	52.6	-26.4
de Geus et al., 2011	240	61	-14
Katagiri et al., 2013	118	-4.5	42.5
Kim et al., 2013	449	61.9	-222.1
Lentini et al., 2012	166	-47	-48
Mårtensson et al., 2015	118	-18.8	28.2
Pickering et al., 2013	66	-11.164	87.836
Ralib et al., 2014	248	40.032	-42.968
Hjortrup et al., 2015	559.1	148.6	-245.5
Breidthardt et al., 2012	73.35	-34.55	57.1
Di Somma et al., 2013	62	-70.8	32.2
Kavalci et al., 2014	297	297	165
Soto et al., 2013	97	2.98	70.98
Doi et al., 2013	102	-2.01	60.99
Perrotti et al., 2015	155.00	-37.97	-27.97
Lipcsey et al., 2014	112	-4	49
Park et al., 2015	96	4.7	73.7
Median (25–75 CI)	118 (96.25–244.0)	-3.0 (-30.61–40.03)	32.6 (-27.58–60.99)

All numbers represent urine or plasma NGAL concentrations in ng/mL; *The meta-analyzed NGAL cutoff concentration for AKI in urine was 81ng/mL, and 165ng/mL for plasma.[1] Formula used in third and fourth column: ([Youden Index, ng/mL]–[mean NGAL concentration, ng7mL]).
Abbreviations: AKI, acute kidney injury; CI, confidence interval; NGAL, Neutrophil gelatinase-associated lipocalin.

S2.6 Mean NGAL concentrations according to RIFLE stages

Descriptive statistics of mean NGAL concentrations separated for those with RIFLE stages risk, injury and failure as opposed to Non-AKI are provided in this chapter.

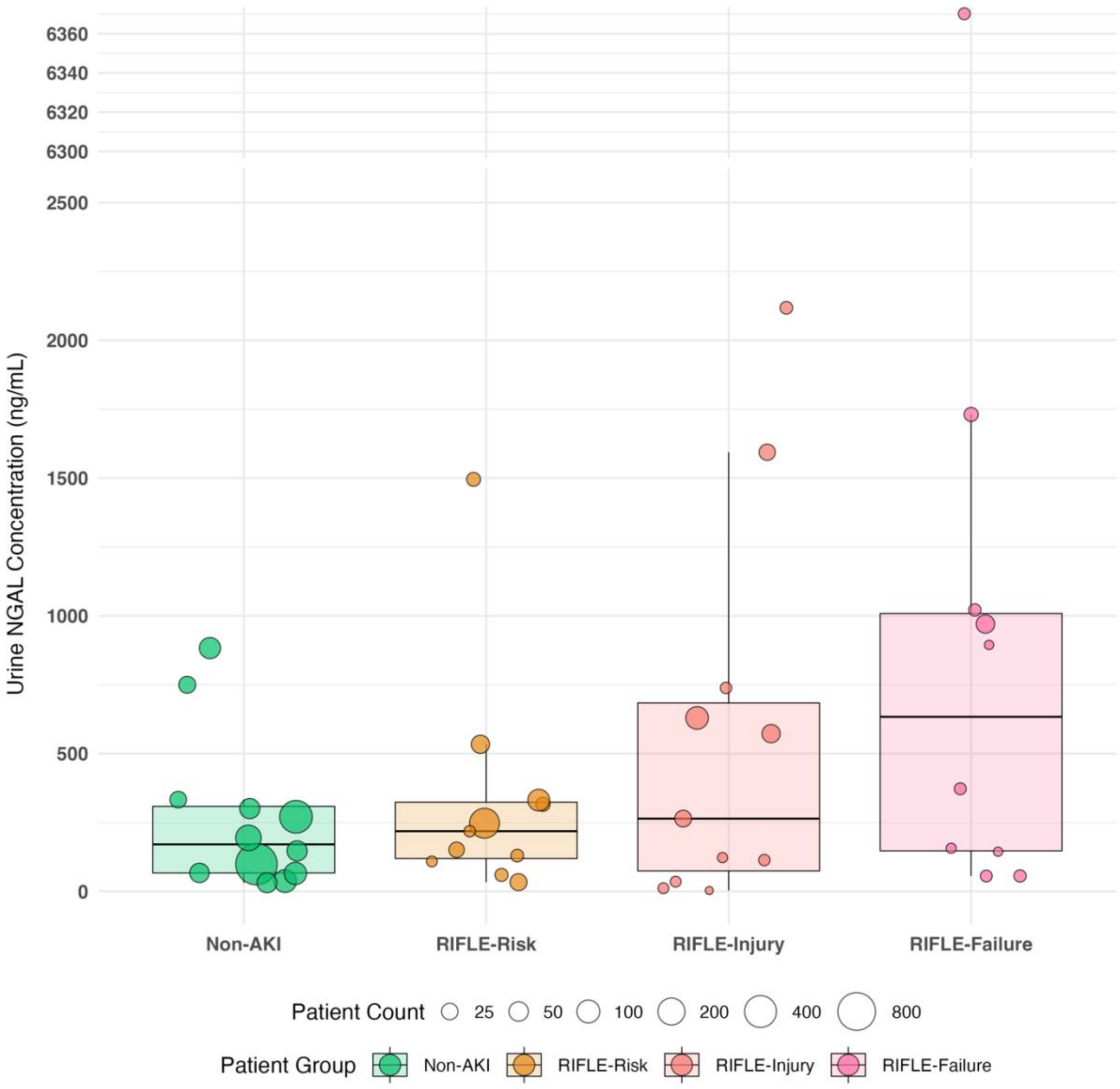

Supplementary Figure 9a: Median and quartiles of N=12 studies' mean urine NGAL concentration for No-AKI and RIFLE stages risk (R), injury (I) and failure (F). Each bubble is representative of the group sample size of each study. Boxes represent the median (25–75th IQR) of all available studies' mean urine and plasma NGAL concentrations; Whiskers represent ±1.5x multiple of IQR. Studies with N≤1 event are left out in the respective RIFLE stage plot as no standard deviation could be calculated.

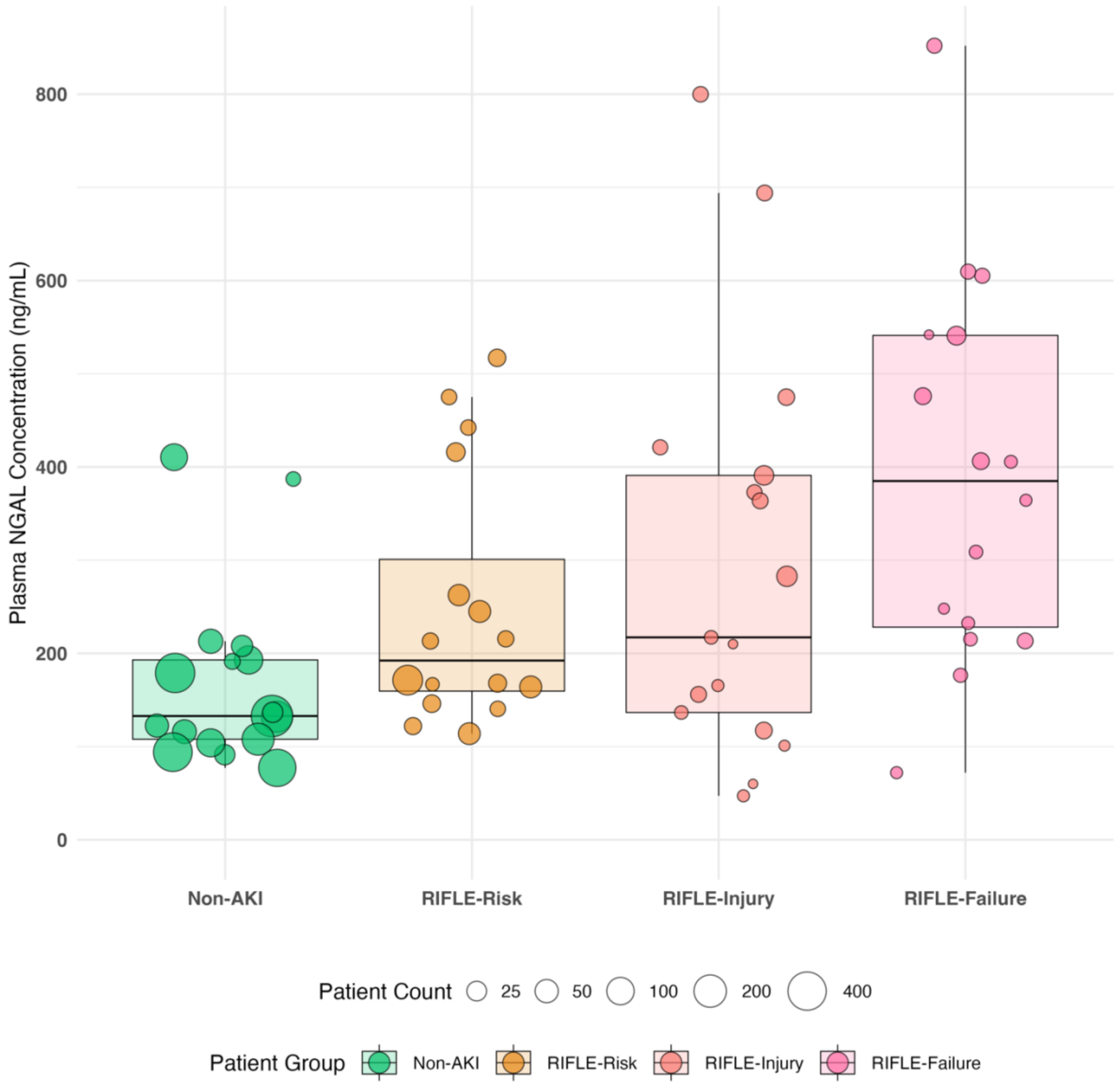

Supplementary Figure 9b: Median and quartiles of N=18 studies' mean plasma NGAL concentration for No-AKI and RIFLE stages risk (R), injury (I) and failure (F). Each bubble is representative of the group sample size of each study. Boxes represent the median (25–75th IQR) of all available studies' mean urine and plasma NGAL concentrations; Whiskers represent ±1.5x multiple of IQR. Studies with N≤1 event are left out in the respective RIFLE stage plot as no standard deviation could be calculated.

Supplementary References

1. Albert C, Zapf A, Haase M, et al. Neutrophil Gelatinase-Associated Lipocalin Measured on Clinical Laboratory Platforms for the Prediction of Acute Kidney Injury and the Associated Need for Dialysis Therapy: A Systematic Review and Meta-analysis. *Am J Kidney Dis*. 2020;76(6):826-841.e1. doi:10.1053/j.ajkd.2020.05.015

2. Sterne JAC, Sutton AJ, Ioannidis JPA, et al. Recommendations for examining and interpreting funnel plot asymmetry in meta-analyses of randomised controlled trials. *BMJ*. 2011;343(jul22 1):d4002. doi:10.1136/bmj.d4002

3. Lau J, Ioannidis JPA, Terrin N, Schmid CH, Olkin I. The case of the misleading funnel plot. *BMJ*. 2006;333(7568):597. doi:10.1136/bmj.333.7568.597

4. Terrin N, Schmid CH, Lau J, Olkin I. Adjusting for publication bias in the presence of heterogeneity. *Stat Med*. 2003;22(13):2113-2126. doi:10.1002/sim.1461

5. Ioannidis JPA, Trikalinos TA. The appropriateness of asymmetry tests for publication bias in meta-analyses: a large survey. *Can Méd Assoc J*. 2007;176(8):1091-1096. doi:10.1503/cmaj.060410

6. Bellomo R, Ronco C, Kellum JA, Mehta RL, Palevsky P, workgroup ADQI. Acute renal failure – definition, outcome measures, animal models, fluid therapy and information technology needs: the Second International Consensus Conference of the Acute Dialysis Quality Initiative (ADQI) Group. *Crit Care*. 2004;8(4):R204-R212. doi:10.1186/cc2872

7. Ostermann M, Zarbock A, Goldstein S, et al. Recommendations on Acute Kidney Injury Biomarkers From the Acute Disease Quality Initiative Consensus Conference. *Jama Netw Open*. 2020;3(10):e2019209. doi:10.1001/jamanetworkopen.2020.19209

Abbreviations

AKI	acute kidney injury
AUC	area under the curve
CI	confidence interval
CS	cardiac surgery
ED	emergency department
ICU	intensive care unit
MD	mean difference
NGAL	neutrophil gelatinase-associated lipocalin
RIFLE	renal risk, injury, failure, loss of renal function, end stage renal disease classification
ROC	receiver operating characteristic.
RRT	renal replacement therapy
SCr	serum creatinine
SD	standard deviation
SE	standard error
UOC	urinary output criteria of the corresponding AKI classification

Authors' affiliations

1. Department of Nephrology, Central Clinic Bad Berka, Bad Berka, Germany
2. University Clinic for Cardiology and Angiology, Otto-von-Guericke University Magdeburg, Magdeburg, Germany
3. Department of Neonatology, Charité University Medicine Berlin, Berlin, Germany
4. Postgraduate School of Emergency Medicine Faculty of Medicine and Psychology University La Sapienza of Rome, Rome, Italy
5. Department of Laboratory Medicine, Konkuk University School of Medicine, Seoul, Republic of Korea
6. Department of Intensive Care, The Austin Hospital, Melbourne, Australia
7. Centre for Integrated Critical Care, The University of Melbourne, Melbourne, Australia
8. Division of Nephrology and Hypertension, Cincinnati Children's Hospital, University of Cincinnati, OH, USA
9. Departments of Internal Medicine, Nephrology and Cardiology, University Hospital Basel, Basel, Switzerland
10. Service de réanimation médicale, Hôpital Saint-André, centre hospitalier universitaire de Bordeaux, Bordeaux, France
11. Department of Thoracic and Cardio-Vascular Surgery, University Hospital Jean Minjoz, Besançon
12. Division of Nephrology and Hypertension, University of California, San Diego, CA, USA
13. Department of Intensive Care, Erasmus University Medical Center, Rotterdam, The Netherlands

14. Department of Emergency and Critical Care Medicine, The University of Tokyo, Hongo, Bunkyo, Tokyo, Japan
15. Department of Nephrology, Prince of Wales Hospital and Clinical School, University of New South Wales, Randwick, Sydney, Australia
16. Department of Anesthesiology, Hospital de la Santa Creu i Sant Pau, Barcelona, Spain
17. Department of Nephrology and Hypertension, Hannover Medical School, Hannover, Germany
18. Medical Faculty, Otto-von-Guericke University Magdeburg, Magdeburg, Germany
19. Department of Cardiology, Immanuel Diakonie Bernau, Heart Center Brandenburg, Brandenburg Medical School Theodor Fontane (MHB), Bernau, Germany
20. Institute of Social Medicine and Health Systems Research, Otto-von-Guericke University Magdeburg, Magdeburg, Germany
21. Department of Intensive Care, Copenhagen University Hospital—Rigshospitalet, Copenhagen, Denmark
22. Department of Cardiothoracic Anaesthesia and Intensive Care, The Heart Center, Copenhagen University Hospital—Rigshospitalet, Copenhagen, Denmark
23. Vascular Unit, First Department of Surgery, “Laiko” General Hospital, Medical School, National and Kapodistrian University of Athens, Athens, Greece
24. Emergency Department, Faculty of Medicine Baskent University, Ankara, Turkey
25. Department of Cardiology and Intensive Care Medicine, Central Clinic Bad Berka, Bad Berka, Germany
26. Medical Faculty, Philipps University of Marburg, Marburg, Germany
27. Department of Nephrology and Dialysis, San Bassiano Hospital, Bassano del Grappa, Italy
28. Center for Cardiology and Angiology, Agaplesion Bethanien Krankenhaus, Frankfurt, Germany
29. Department of Cardiology, Kerckhoff Clinic, Bad Nauheim, German
30. CIRRUS, Hedenstierna laboratory, Anaesthesiology and Intensive care, Department of Surgical Sciences, Uppsala University, Uppsala, Sweden
31. Section of Anaesthesia and Intensive Care Medicine, Department of Physiology and Pharmacology, Karolinska Institutet, Stockholm, Sweden
32. First Critical Care Department, 'Evangelismos' General Hospital, National and Kapodistrian University of Athens, Athens, Greece
33. Department of Medicine, Division of Nephrology, Columbia University, New York, NY, USA
34. Department of Emergency Medicine, Christchurch Hospital, Christchurch, New Zealand
35. Department of Medicine, University of Otago Christchurch, Christchurch, New Zealand
36. Medical Faculty University of Padova and Department of Nephrology, Dialysis & Transplantation, International Renal Research Institute Vicenza (IRRIV), San Bortolo Hospital, Vicenza, Italy
37. Department of Nephrology, Dialysis and Transplantation, Hospital Italiano de Buenos Aires, Buenos Aires, Argentina
38. Department of Anaesthesiology and Intensive Care, International Islamic University Malaysia, Kuantan, Pahang, Malaysia
39. Department of Nephrology, Hospital Fernando Fonseca, Lisbon, Portugal
40. Department of Medical Biometry and Epidemiology, University Medical Center Hamburg-Eppendorf, Hamburg, Germany
